# The molecular chaperone GRP170 protects against ER stress and acute kidney injury in mice

**DOI:** 10.1172/jci.insight.151869

**Published:** 2022-03-08

**Authors:** Aidan W. Porter, Diep N. Nguyen, Dennis R. Clayton, Wily G. Ruiz, Stephanie M. Mutchler, Evan C. Ray, Allison L. Marciszyn, Lubika J. Nkashama, Arohan R. Subramanya, Sebastien Gingras, Thomas R. Kleyman, Gerard Apodaca, Linda M. Hendershot, Jeffrey L. Brodsky, Teresa M. Buck

**Affiliations:** 1 Department of Biological Sciences,; 2 Department of Pediatrics, Nephrology Division,; 3 Department of Medicine, Renal-Electrolyte Division,; 4 Department of Immunology,; 5 Department of Cell Biology, and; 6 Department of Pharmacology and Chemical Biology, University of Pittsburgh, Pittsburgh, Pennsylvania, USA.; 7 Department of Tumor Cell Biology, St. Jude Children’s Research Hospital, Memphis, Tennessee, USA.

**Keywords:** Nephrology, Cell stress, Chaperones, Mouse models

## Abstract

Molecular chaperones are responsible for maintaining cellular homeostasis, and one such chaperone, GRP170, is an endoplasmic reticulum (ER) resident that oversees both protein biogenesis and quality control. We previously discovered that GRP170 regulates the degradation and assembly of the epithelial sodium channel (ENaC), which reabsorbs sodium in the distal nephron and thereby regulates salt-water homeostasis and blood pressure. To define the role of GRP170 — and, more generally, molecular chaperones in kidney physiology — we developed an inducible, nephron-specific GRP170-KO mouse. Here, we show that GRP170 deficiency causes a dramatic phenotype: profound hypovolemia, hyperaldosteronemia, and dysregulation of ion homeostasis, all of which are associated with the loss of ENaC. Additionally, the GRP170-KO mouse exhibits hallmarks of acute kidney injury (AKI). We further demonstrate that the unfolded protein response (UPR) is activated in the GRP170-deficient mouse. Notably, the UPR is also activated in AKI when originating from various other etiologies, including ischemia, sepsis, glomerulonephritis, nephrotic syndrome, and transplant rejection. Our work establishes the central role of GRP170 in kidney homeostasis and directly links molecular chaperone function to kidney injury.

## Graphical abstract

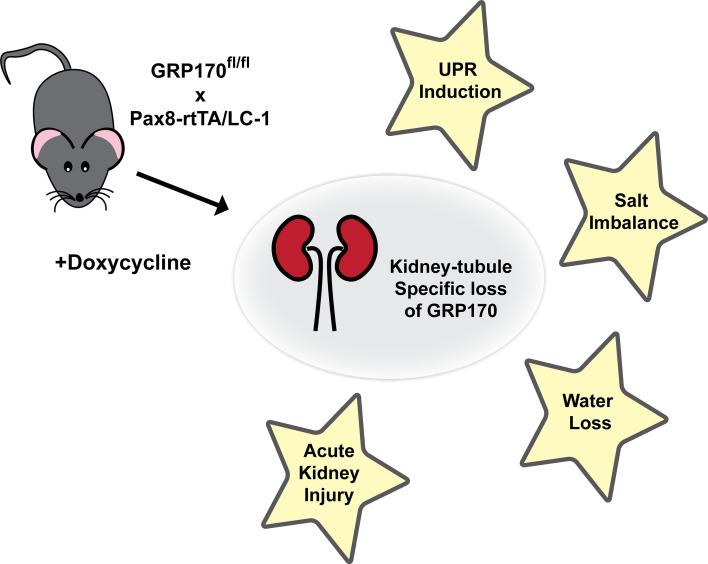

## Introduction

The kidneys maintain salt and water homeostasis, reclaim nutrients, and eliminate waste. These critical functions are accomplished by ion channels and solute transporters expressed along the length of the nephron from the proximal tubule (PT) to the collecting duct (CD). To respond to environmental changes, renal ion channels and transporters are subject to transcriptional and posttranslational regulation. Posttranslational regulatory mechanisms govern protein folding, chemical modifications, protein trafficking through the secretory pathway, and degradation (1–4). Each of these critical posttranslational events is orchestrated by molecular chaperones. In contrast to our understanding of how molecular chaperones support these events, how members of this conserved protein family maintain organismal homeostasis and protein homeostasis (i.e., “proteostasis”) is understudied.

Molecular chaperones are especially vital to promote the folding of proteins in the endoplasmic reticulum (ER). In the event that folding proceeds inefficiently in this compartment, many of these same chaperones select and then deliver misfolded proteins for degradation (5–8). Perhaps not surprisingly, there are numerous links between molecular chaperone function and ion channels and transporters in the kidney. For example, multichaperone complexes interact with the thiazide-sensitive NaCl cotransporter (NCC), the epithelial sodium channel (ENaC), the renal outer medullary potassium channel (ROMK), the sodium-potassium ATPase, and the sodium-potassium chloride cotransporter-2 (NKCC2) to facilitate protein folding, to promote proteosome-mediated degradation of misfolded forms of these proteins, and to ensure trafficking of these substrates within the cell (8–13). The ability of select molecular chaperones to orchestrate protein biogenesis is critical because defective ion channel and transporter biogenesis and/or function due to inherited mutations in ion channels results in several renal diseases, such as pseudohypoaldosteronism type-1 and Liddle syndrome (ENaC), Gitelman syndrome (NCC), and Bartter syndrome (NKCC2, NCC, and ROMK) (14–17). Despite the importance of select chaperones in renal physiology, the roles of most molecular chaperones in the kidney are also uncharacterized.

One such molecular chaperone, GRP170 (also known as ORP150 and HYOU1), is an abundant, ER-localized Hsp70-like protein. Hsp70 chaperones contain nucleotide- and substrate-binding domains (NBD and SBD, respectively) that couple ATP hydrolysis to the binding and release of protein substrates. GRP170 also harbors SBD extensions that may modify substrate specificity (18–20). In addition, GRP170, as well as the yeast homolog, Lhs1, regulate the ATPase activity of a bona fide ER-resident Hsp70, BiP (21–23). We previously determined that GRP170 directly facilitates the proteasome-dependent, ER-associated degradation (ERAD) of unassembled ENaC subunits in yeast*,* mammalian cell lines, and *Xenopus* oocytes (8, 24, 25). These data suggest that GRP170 helps maintain ER proteostasis. Consistent with this model, GRP170 is cytoprotective when renal tubule epithelial cells are exposed to hypoxic and hyperosmolar stress (26). Moreover, the loss of GRP170 is embryonically lethal in rodents, and heterozygous KO animals are more susceptible to ischemic injury than WT controls or GRP170-overexpressing littermates (26). Nevertheless, it is unknown whether GRP170 maintains essential kidney-dependent homeostasis in animals.

Like most other molecular chaperones in the ER, GRP170 expression is induced by the unfolded protein response (UPR). The UPR maintains ER proteostasis, and when unmitigated, it triggers cell death (27, 28). Notably, the UPR has also been linked to renal disorders, including ischemic injury, diabetic and contrast nephropathy, glomerulonephritis, and congenital and acquired nephrotic syndrome (29–33). Despite the established importance of the UPR in tempering ER stress, the relationship between the functions of most specific molecular chaperones and proteostasis is unclear. Furthermore, how ER stress contributes to the deterioration of renal function and cellular physiology — and how molecular chaperones might mitigate the consequences of ER stress in the kidney — has never been comprehensively investigated in vivo. Recent discoveries found that therapeutics can mitigate ER stress and regulate molecular chaperone levels (34, 35). Therefore, defining the mechanisms that link molecular chaperone function to renal cell proteostasis may reveal novel treatments for renal injury, most notably acute kidney injury (AKI). Indeed, drugs that augment ER proteostasis show promise in clinical trials for Alzheimer’s disease and ALS (36, 37, 38).

AKI is the abrupt deterioration of renal function that manifests as the decreased clearance of metabolic waste products and toxic metabolites, as well as the disruption of electrolyte and water homeostasis. AKI is a common life-threatening condition, affecting 10%–15% of all hospitalized patients and approximately half of all ICU admissions (39). AKI predicts not only increased in-hospital morbidity and mortality (40–42), but it also predisposes patients to chronic kidney disease (CKD) (43), hypertension (44), and the need for renal replacement therapy (45). Despite its prevalence and adverse sequela, AKI remains an intractable condition with clinicians’ options limited to prevention and the management of complications while awaiting spontaneous renal recovery (46). Effective AKI treatments are lacking, in part, because of the spectrum of disorders and, hence, diverse pathophysiologic conditions that undergird its development. For example, systemic illnesses, such as sepsis and vasculitis, exhibit different clinical and molecular features than more localized conditions, such as ischemia-reperfusion (IR), pyelonephritis, or urinary tract obstruction (46). Ultimately, most cases of AKI are triggered by an environmental stressor, including sepsis, hypoxia, aberrant oxygen metabolism, and nutrient depletion, that disrupts homeostasis and more specifically ER proteostasis (47). Therefore, deficits in the function of ER chaperones may contribute to AKI.

To delineate the relationship between molecular chaperone function, ER proteostasis, and nephron physiology, we generated an inducible, kidney-tubule specific, GRP170-KO mouse. We demonstrate that GRP170 deficiency results in a severe, rapidly degenerating phenotype: dramatic weight loss, electrolyte wasting, and AKI. We also found that the loss of GRP170 triggers the UPR. Our work not only positions GRP170 in the maintenance of renal proteostasis in vivo, but it also provides a potentially new model, one in which secondary insults and therapeutics that offset the catastrophic consequences of AKI can be investigated.

## Results

### Construction of an inducible, nephron-specific GRP170-KO mouse.

**Figure 1:**
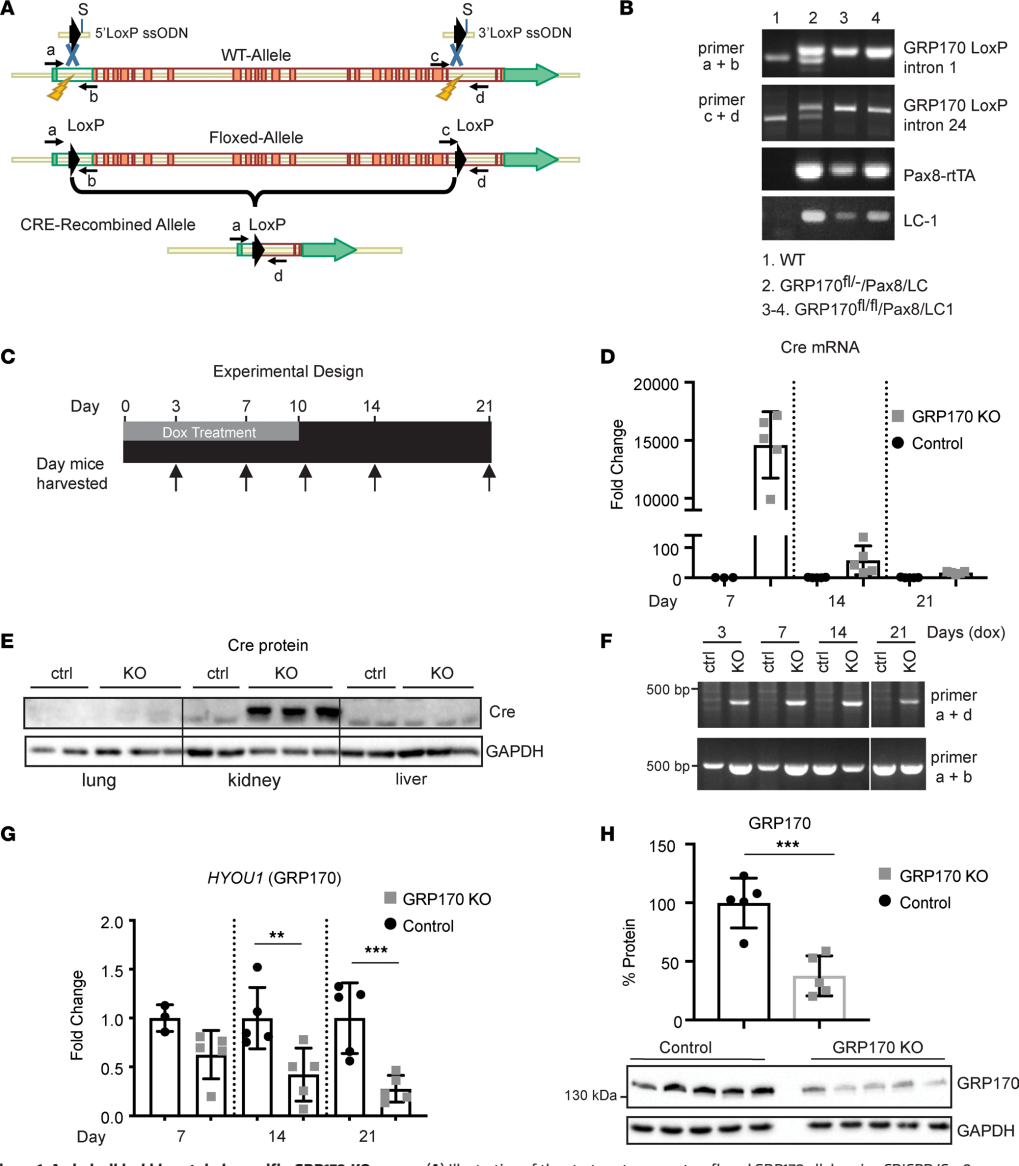
An inducible, kidney tubule specific, GRP170-KO mouse. (**A**) Illustration of the strategy to generate a floxed GRP170 allele using CRISPR/Cas9 technology. Location of single-stranded oligo donors (ssODN) primers used for genotyping are noted (a, b, c, d). (**B**) PCR analysis from tail samples was used to confirm the genotype of WT, control, and experimental animals. (**C**) Experimental timeline used to study the GRP170-KO mice. (**D** and **E**) qPCR or Western blot analysis (day 21) of kidney lysates for Cre recombinase expression following Dox treatment was performed for the indicated times in indicated tissues. GAPDH served as a loading control. (**F**) A PCR analysis with primers a and d (designated in **A**) using whole kidney lysates from either control (lanes 1–5) or experimental (lanes 6–10) mice treated with Dox was performed to confirm genetic editing. (**G** and **H**)Mice were sacrificed on the indicated days. Kidney lysates from control or GRP170-KO mice were subject to either qPCR (**G**) or SDS-PAGE and Western blotting (**H**) to detect GRP170 levels (western blots from day 14 lysates). In **H**, blots were probed with anti-GRP170 antibody or GAPDH as a loading control. Data presented are the means ± SD; *P < 0.05, **P < 0.01, ***P < 0.001, ****P < 0.0001. Statistical significance determined by 1-way ANOVA followed by Tukey’s multiple-comparison test (3 or more data sets) or 2-tailed Student’s *t* test (2 data sets). Data presented are the means ± SD.

Because a germline GRP170-KO mouse is embryonically lethal (48), we developed an inducible, kidney-tubule specific GRP170-KO mouse using the characterized *Pax8*-rtTA/LC-1 system (49, 50). The gene encoding GRP170, HYOU1, contains 26 exons and a start codon in exon 2, so LoxP sites were introduced into introns 1 and 24 to ultimately remove the majority of the coding sequence using Cas9 and a pair of sgRNAs to induce double-strand breaks (Figure 1A). PCR genotyping identified correctly inserted LoxP sites (Figure 1B), which we confirmed by TOPO cloning and sequencing (data not shown) (Supplemental Methods; supplemental material available online with this article; https://doi.org/10.1172/jci.insight.151869DS1). The resulting GRP170^fl/fl^ mice were then crossed to a *Pax8*-rtTA/LC-1 mouse to create triple-transgenic GRP170^fl/fl^/*Pax8*/LC-1 (hereafter referred to as GRP170-KO) animals homozygous for the GRP170-floxed allele and containing at least 1 copy of the Pax8-rtTA (Pax8) and LC-1 alleles. The triple transgenic mouse was confirmed by PCR genotyping of tail samples (Figure 1B). *Pax8* specifically drives expression of the reverse tetracycline-dependent transactivator (rtTA) along the embryonic and mature nephron, while sparing the glomerulus (49, 51–53). In turn, doxycycline (Dox) binds rtTA to induce the expression of Cre recombinase from LC-1 (P_tet_-Cre) (54). Therefore, the specificity of Cre recombinase expression was verified by first crossing the Pax8/LC-1 strain to the Rosa26/CAG^TdTomato^ reporter strain, which expresses the red fluorescent protein, TdTomato, in response to Cre recombinase expression. Consistent with extensive analyses from other groups (49, 55, 56), we found that Cre expression was present in all tubule segments but excluded from glomeruli (Supplemental Figure 1).

GRP170-KO mice and transgenic GRP170^fl/fl^/*Pax8* or GRP170^fl/fl^/LC-1 littermates (hereafter referred to as the controls) were next treated with Dox (0.2 mg/mL) in drinking water for up to 10 days to induce Cre expression and, thus, GRP170 genetic ablation (Figure 1C). To monitor the expression of Cre recombinase, we performed quantitative PCR (qPCR) and Western blot analysis of whole kidney lysates. Robust Cre message was detected at day 7, but not at time points after Dox administration ceased (days 14 and 21; Figure 1D). As expected, Cre was expressed in the kidney but was undetectable in lung and liver (Figure 1E) (49, 51–53). To confirm regulated and prompt genetic editing, PCR was performed on DNA extracts from whole kidney lysates to amplify an edited-specific product (i.e., oligo pair a + d; Figure 1A). By day 3, this product was detected in GRP170-KO but not control specimens (Figure 1F). We also found a time-dependent decrease in GRP170 mRNA and protein levels in the GRP170-KO animals (Figure 1, G and H, and data not shown). As anticipated, residual GRP170 message and protein was apparent because whole kidney lysates rather than isolated renal tubules (where Cre recombinase was specifically expressed) were used. Based on these data, in subsequent experiments, Dox was administered for 10 days unless otherwise noted.

### GRP170-KO mice exhibit volume loss and natriuresis.

**Figure 2:**
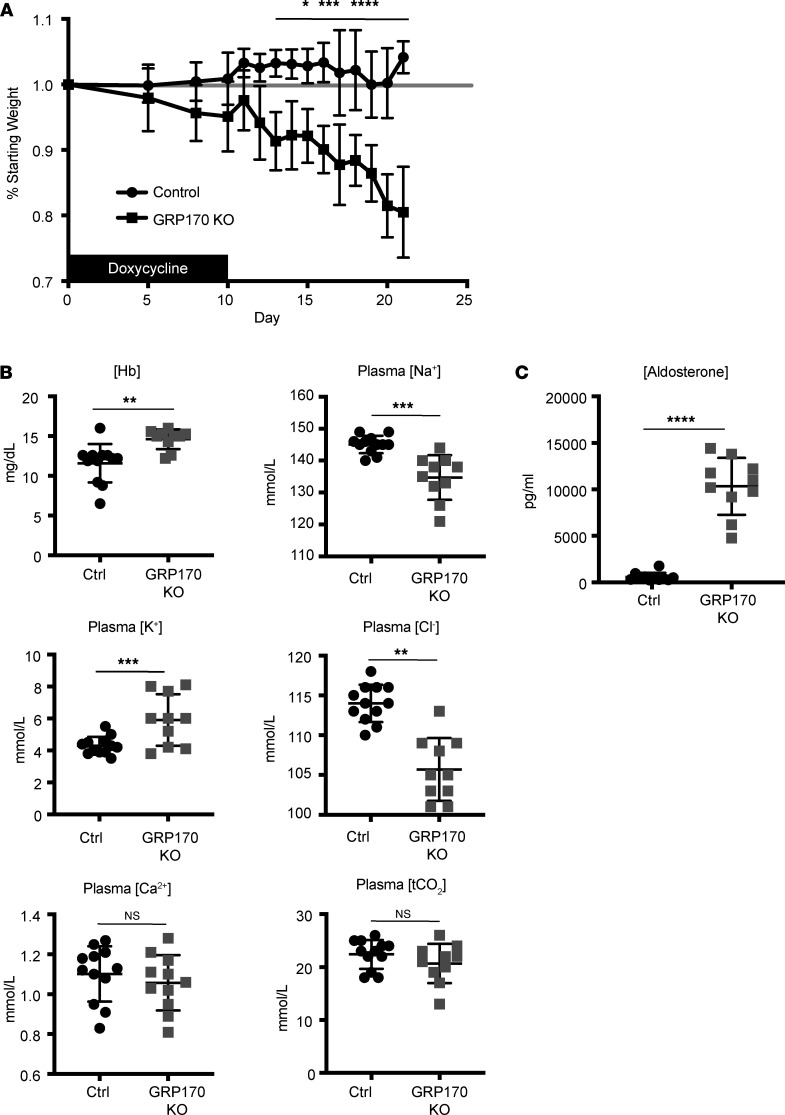
The GRP170-KO mice exhibit volume loss. (**A**) Dox was administered to GRP170-KO and control mice for 10 days, and weights were monitored as indicated (shown as mean ± SD; *n* = 10–12). Weights from GRP170-KO mice were significant at the following days: *P < 0.05 (day 14), ***P < 0.001 (day 17, 18, 19), ****P < 0.0001 (day 15, 16, 19, 20). (**B** and **C**) Plasma electrolytes (**B**) and aldosterone (**C**) were measured on day 21 following Dox administration as described in the Methods. Data represent the means ± SD; *n* = 10–12; **P < 0.01, ***P < 0.001, ****P < 0.0001. Statistical significance determined by 1-way ANOVA followed by Tukey’s multiple-comparison test (3 or more data sets) or 2-tailed Student’s *t* test (2 data sets). Data presented are the means ± SD.

The potential role of GRP170 — and, more generally, ER molecular chaperones — in supporting nephron function in an inducible system has never before been investigated. Because this system allows for real-time measurements of ER proteostasis and animal physiology as a chaperone is depleted, we first monitored weight and blood chemistry of the GRP170-KO mice and littermate controls. As shown in Figure 2A, the GRP170-KO mice had lost significant weight by day 14 after Dox treatment began, in contrast to the control animals. By day 21, the KO animals lost ~20% of bodyweight and had elevated plasma hemoglobin, consistent with hemoconcentration from depletion of extracellular fluid volume (Figure 2, A and B). The GRP170-KO animals were also hyponatremic, hypochloremic, and hyperkalemic, but they maintained normal plasma calcium and bicarbonate levels (Figure 2B). Because hypovolemia and concomitant hyponatremia and hyperkalemia indicate a combination of impaired sodium, potassium, and water handling — and because these abnormalities stimulate renin, angiotensin, and aldosterone secretion — we anticipated that plasma aldosterone would also rise (57, 58). Strikingly, the average plasma aldosterone concentration in GRP170-KO animals was > 17-fold that of control animals (Figure 2C). By comparison, loss of αENaC in the mouse nephron results in an ~11-fold increase in plasma aldosterone (56), and loss of SGK1, a positive regulator of ENaC, resulted in only a ~2-fold increase (59).

**Figure 3:**
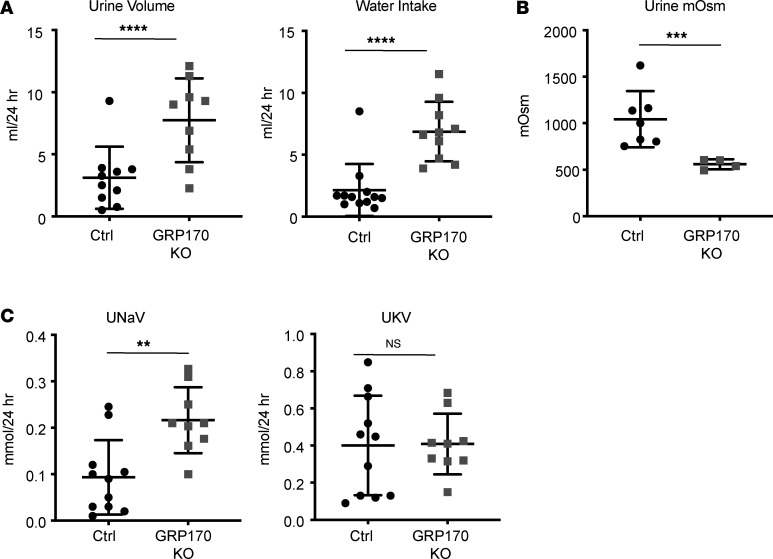
GRP170 deficiency increases urine volume and results in natriuresis. (**A**–**C**) GRP170-KO and control mice were placed in individual metabolic cages to measure 24-hour urine volume and water intake (**A**), urine osmolality (**B**), and urine sodium and potassium (**C**). Osmolality was measured with an osmometer and sodium and potassium were quantified after flame photometry. Data represent the means ± SD; *n* = 10-12; **P < 0.01, ***P < 0.001, ****P < 0.0001. Statistical significance determined by 1-way ANOVA followed by Tukey’s multiple-comparison test (3 or more data sets) or 2-tailed Student’s *t* test (2 data sets). Data presented are the means ± SD.

We next measured urine output and water intake of animals housed in individual metabolic cages. GRP170-KO animals displayed both significantly increased 24-hour urine output and water intake (Figure 3A). As anticipated, the urine of KO mice was also significantly more dilute than that of control animals (Figure 3B), and urine solute and electrolyte analysis established that GRP170-KO mice inappropriately excrete sodium. Potassium secretion was, however, comparable with that of controls (Figure 3C), an observation that may reflect the effect of flow-dependent potassium secretion and/or the establishment of a new, compensatory equilibrium for potassium intake and excretion. These data also suggest that GRP170 function has roles in the regulated reabsorption of filtered sodium and urinary concentration in the mouse nephron. Interestingly, this phenotype is akin to that observed when an inducible, nephron-specific αENaC-KO mouse was examined (56). In that study, deletion of αENaC led to rapid weight loss, natriuresis, hyperkalemia, and significantly elevated aldosterone levels, as noted above. In accordance with a phenotype that mirrors the loss of αENaC, our previous work demonstrated that GRP170 regulates the levels of unassembled ENaC subunits and the trafficking of this channel to the cell surface (8, 25).

### Loss of GRP170 damages the PT epithelium and alters ion channel and transporter levels.

The data in the preceding section suggest that GRP170 deficiency affects ion channel and solute transporter levels and/or activity. Another nonmutually exclusive alternate scenario is that the loss of this ER chaperone triggers a stress response that compromises general cell structure and function. In fact, higher aldosterone levels in our model compared with the nephron-specific αENaC-KO mouse (56) might reflect the fact that the toxicity arising from GRP170 deficiency is more closely linked to proteostasis than ion channel loss, although it is also likely that both effects contribute to renal damage.

**Figure 4:**
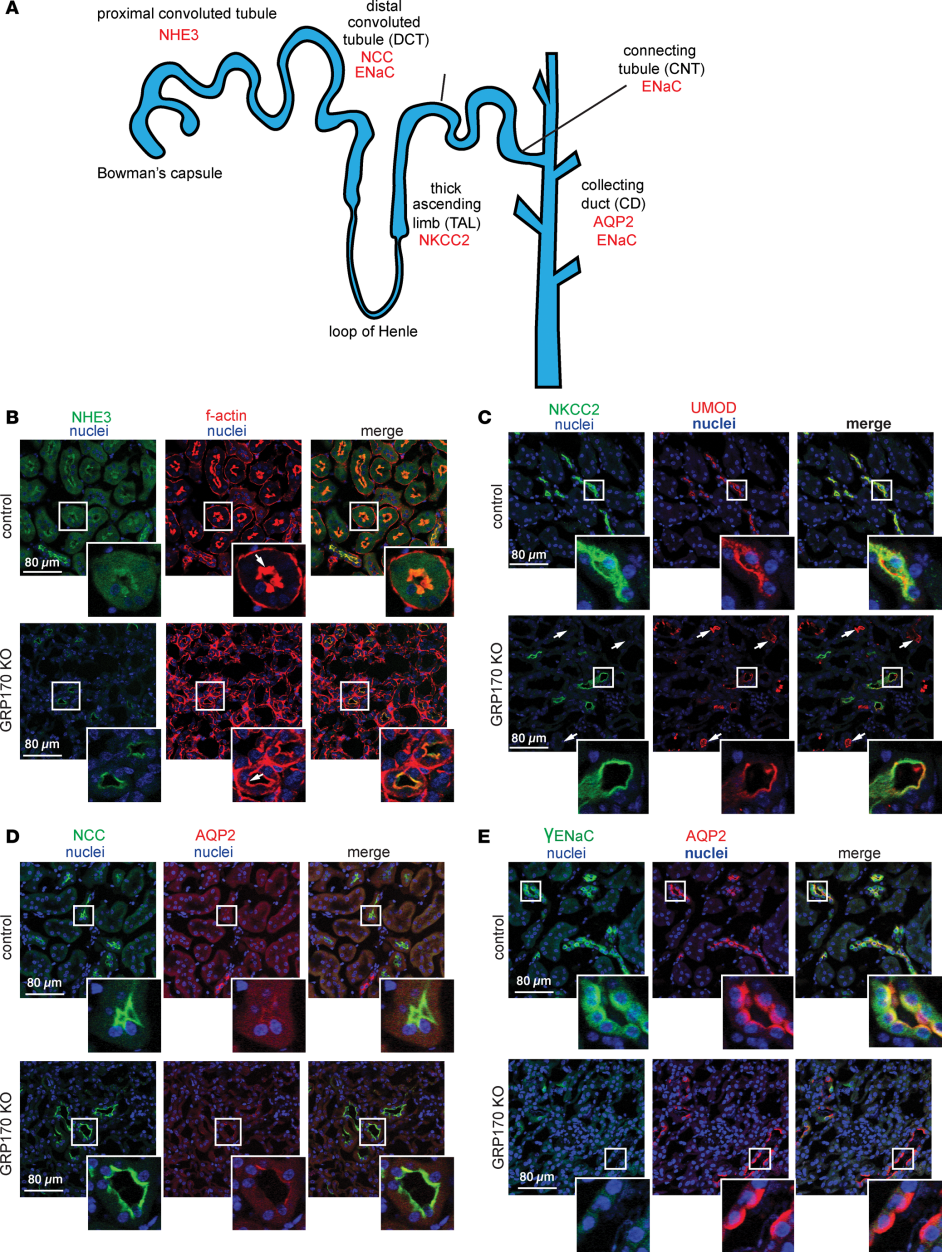
Expression of ion channels and transporters in the GRP170-KO mouse. (**A**–**E**) Model of an individual kidney nephron with the location of select ion channels and transporters indicated. Immunofluorescent localization was performed at day 21 as described in Methods using the following antibodies: anti-NHE3 in the proximal tubule (F-actin is stained by phalloidin and serves as a control) (**B**); anti-NKCC2 (in the TAL) and anti-uromodulin (UMOD), which served as a control (**C**); anti-NCC (in the DCT) and anti-AQP2 (in the CD), which served as a negative control (**D**); and anti-γENaC and anti-AQP2 (which served as a control) (**E**). Scale bar: 80 µm.

To begin to test these predictions, we first assessed the residence and expression of ion channels and transporters along the length of the nephron, namely from the PT to the CD (Figure 4A). We first found that the Na/H exchanger, NHE3, which normally resides in the PT, appears to have diminished expression but remains at the apical membrane in GRP170-KO animals (Figure 4B, left panel). In contrast, phalloidin staining of F-actin — which was used to visualize the prominent brush border surface and identify PT segments — was disrupted. The KO animals instead displayed less prominent apical F-actin staining than controls animals (Figure 4B, compare white arrows). In addition, the normally cuboidal epithelium of the PT instead has a flattened appearance more closely resembling a simple squamous epithelium in the GRP170-KO mice. Similarly, the Na-K-2Cl cotransporter (NKCC), normally expressed in the thick ascending limb (TAL) of the loop of Henle, colocalized with a TAL marker (uromodulin) in control animals, as expected, but in KO mice the majority of tubules (marked by white arrows) display uromodulin staining but lack NKCC (Figure 4C). Although the DCT also expresses uromodulin, but not NKCC, we were unable to observe any such tubules in the control animals. In contrast, the thiazide sensitive Na^+/^Cl^–^ cotransporter (NCC) and aquaporin-2 channel (AQP2), expressed in the distal convoluted tubule (DCT) and in the CD, respectively, were unaffected in GRP170-KO animals and, as expected, did not colocalize (Figure 4D). Finally, consistent with the regulatory role of GRP170 in ENaC biosynthesis (see above), the levels of γENaC (expressed in both the DCT and the CD) were lower in GRP170-KO mice (Figure 4E). Nevertheless, residual γENaC colocalized with AQP2 as expected (Figure 4E). These data indicate that the loss of GRP170 disrupts the trafficking and stability of select ion channels and transporters and alters tubule architecture, particularly of the more proximal nephron segments.

**Figure 5:**
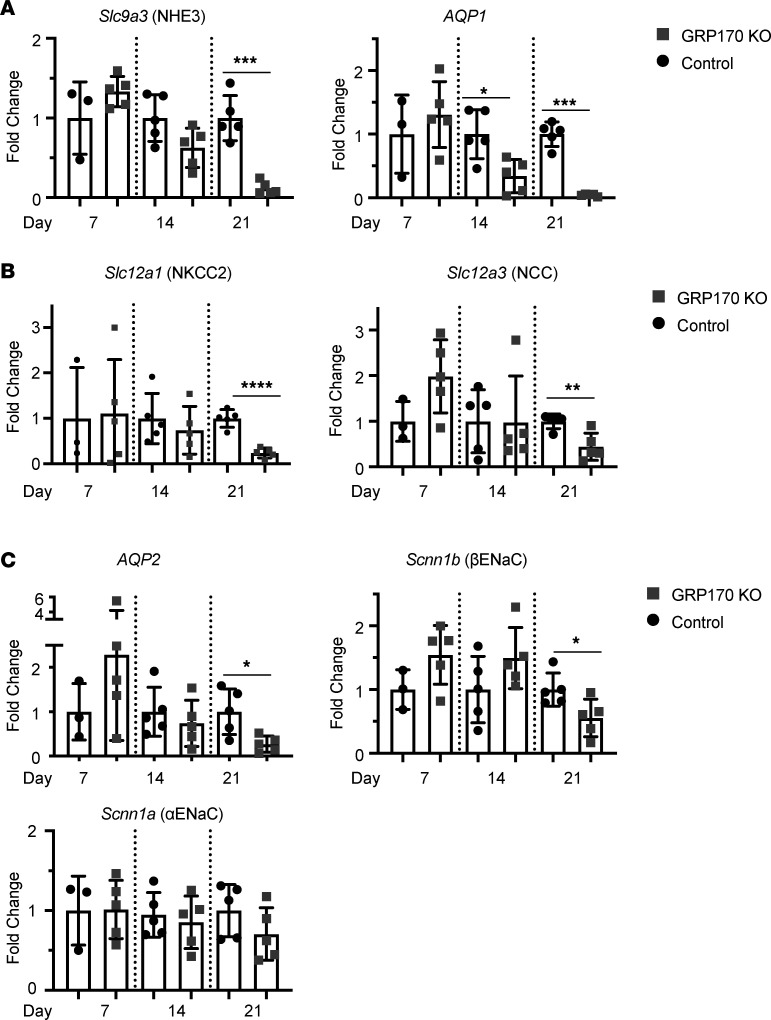
The loss of GRP170 results in a generalized reduction in ion channel/transporter mRNA across the nephron. (**A**–**C**) qPCR was performed on kidney lysates as described in Methods to detect *NHE3* and *AQP1* (proximal tubule) (**A**), *NKCC2* (TAL) and *NCC2* (DCT) (**B**), and ENaC subunits and *AQP2* (collecting duct) (**C**). Data were corrected to actin mRNA and represent the means ± SD (*n* = 5, with the exception of the day 3 control [*n* = 3]) and were analyzed by pairwise comparison using a Student’s *t* test. *P < 0.05, **P < 0.01, ***P < 0.001, ****P < 0.0001. Statistical significance determined by 1-way ANOVA followed by Tukey’s multiple-comparison test (3 or more data sets) or 2-tailed Student’s *t* test (2 data sets). Data presented are the means ± SD.

To more precisely define how GRP170 deficiency affects the levels of channels and transporters in each segment of the nephron, we performed qPCR analysis from whole kidney lysate and normalized values to actin mRNA. Consistent with the more profound effects on proximal regions (Figure 4), mRNA levels corresponding to the proximal tubular transporter, AQP1 (*AQP1*) were reduced by day 14 (Figure 5A), although NHE3 levels (*Slc9a3*) were not. mRNAs for channels located in the more distal nephron segments and CD, αβγENaC and AQP2, were minimally affected (Figure 5C) in the KO animals. Based on the high plasma aldosterone levels of the GRP170-deficient mice (Figure 2), we expected but did not observe an increase in αENaC message. This result suggests that the loss of GRP170 impairs the response to aldosterone, perhaps by altering cellular physiology or the activities/levels of components in the aldosterone signaling pathway. We also noted that the messages encoding the NKCC2 (*Slc12a1*) and NCC (*Slc12a3*) proteins, which are expressed in the loop of Henle and DCT, respectively, were also reduced (Figure 5B). By day 21, there was reduced expression of multiple transporters across the nephron. Based on these combined results, GRP170 expression helps maintain PT function and integrity, which may reflect the higher metabolic demands — and, thus, the increased susceptibility to injury — of this segment (60).

### Loss of GRP170 results in kidney injury and hallmarks of AKI.

**Figure 6:**
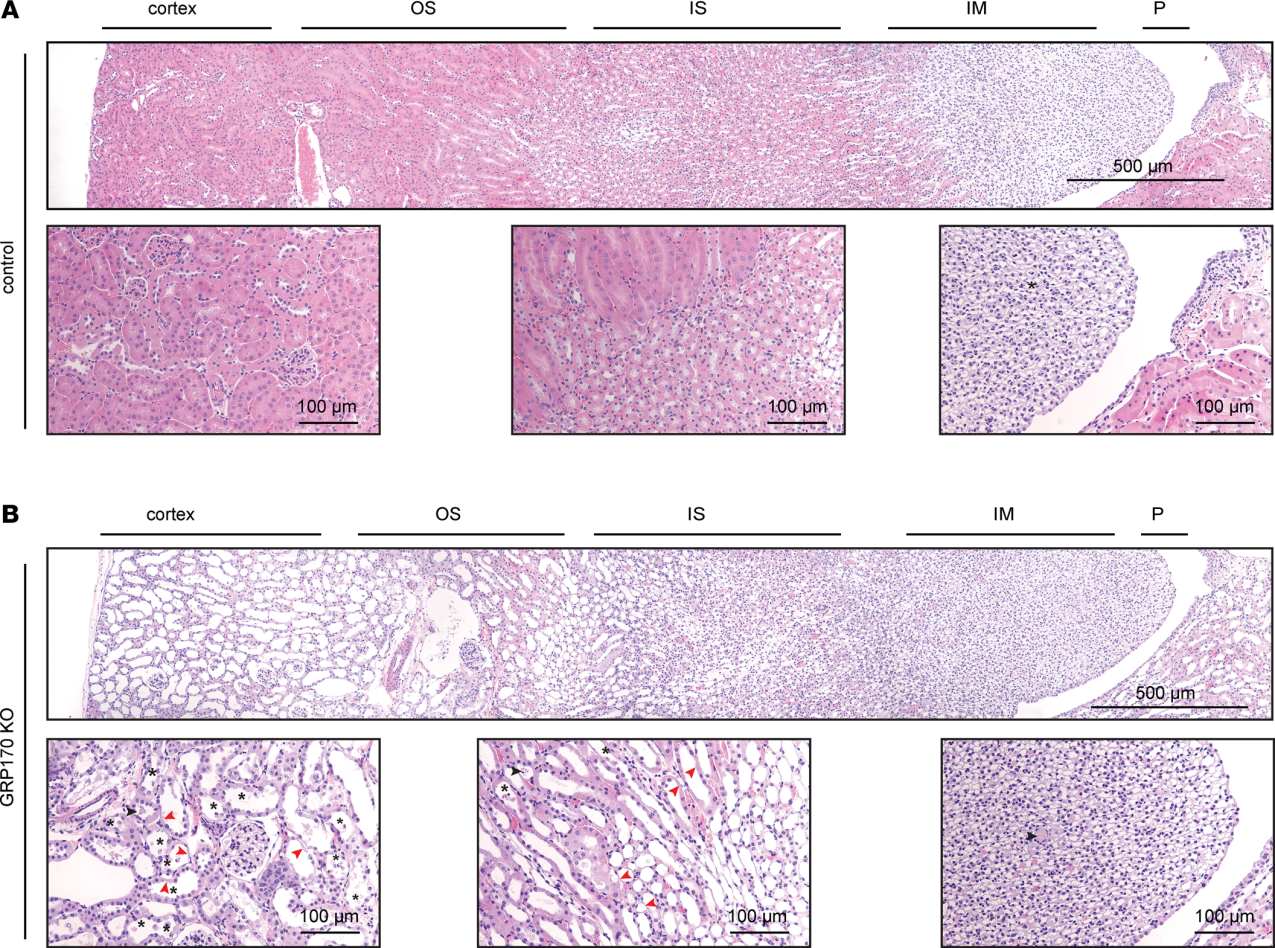
The loss of GRP170 results in widespread kidney tubule dilation and kidney damage. (**A** and **B**) H&E staining of fixed kidney slices from either control (**A**) or GRP170-KO animals (**B**) harvested at day 21 from the start of Dox administration. The following kidney injury–related observations were noted: thinning of epithelial cells and loss of tubule cells (red arrows), sloughing of epithelial cells into the tubule lumen (black asterisks), and granular material/casts (black arrows). Scale bars: 500 µm and 100 µm.

Due to the noted disturbances in PT architecture when GRP170 was depleted, we performed H&E staining on kidney slices from control and GRP170-KO mice sacrificed at day 21. The KO mice exhibited classic histopathological findings of AKI, with widespread and significant tubule dilation and reduced tubular cell volume (compare Figure 6, A and B). Loss of visible brush border (Figure 4B), as well as thinning of epithelial cells and rarefication of tubule cells (Figure 6B, red arrowheads), were also pervasive. Sloughing of epithelial cells and the presence of necrotic cells in the tubule lumen were additionally observed in the GRP170-KO animals (Figure 6B, black asterisks). We also noted an occasional tubule containing granular casts within the tubular lumen (Figure 6, black arrowheads). Interestingly, there were no signs of damage to glomeruli or blood vessels, arguing against concomitant glomerular or vascular injury. Consistent with the data in Figure 5, mice sacrificed at day 7 or 14 displayed an intermediate, progressive phenotype. At day 7, only isolated tubule dilation in the inner medulla was noted; however, by day 14, generalized tubule dilation was discernable in all regions of the kidney and was particularly evident in the cortex. Moreover, trichrome staining of kidney slices from days 7, 14, and 21 demonstrates the clear progression of kidney injury (Supplemental Figure 2).

**Figure 7:**
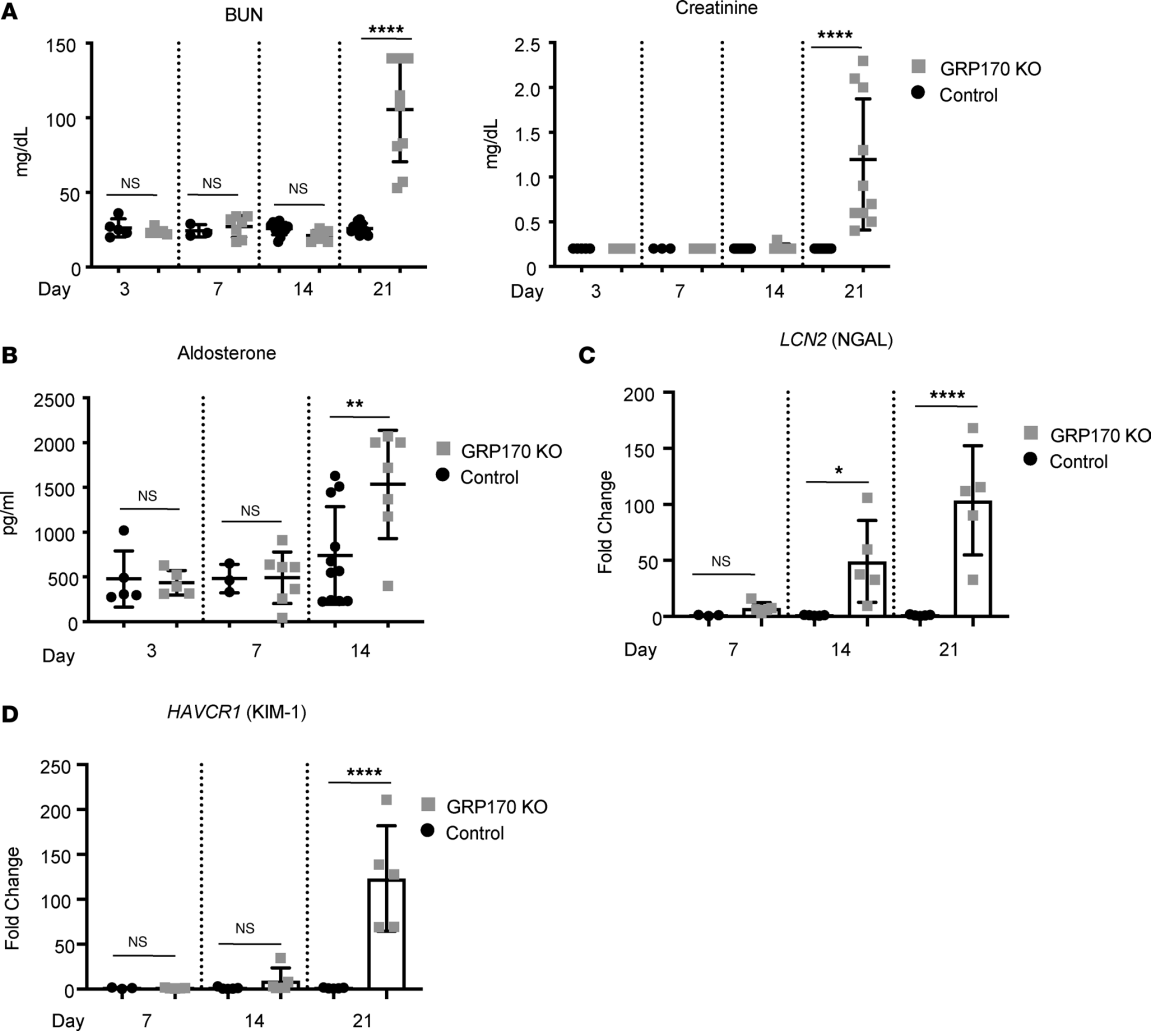
Nephron-specific GRP170 deficiency results in acute kidney injury. (**A** and **B**) BUN, creatinine, and aldosterone levels in plasma samples from either control or GRP170-KO animals sacrificed at day 7, 14, or 21 were measured (*n* = 10–12). (**C** and **D**) NGAL and KIM-1 message was subject to qPCR from control and GRP170-KO kidneys on the indicated day as described in Methods. Data shown represent the means ± SD (*n* = 5, with the exception of the day 7 control [*n* = 3]); *P < 0.05, **P < 0.01, ****P < 0.0001. Statistical significance determined by 1-way ANOVA followed by Tukey’s multiple-comparison test (3 or more data sets) or 2-tailed Student’s *t* test (2 data sets). Data presented are the means ± SD.

Based on these data, we next quantified specific AKI markers in the GRP170-KO mouse. Two functional biomarkers of kidney injury, plasma creatinine and BUN, were significantly elevated in GRP170-KO mice by day 21 (Figure 7A). It is also possible that the increase in creatinine and BUN reflects the volume-depleted state of the GRP170-KO mouse. The levels of these markers were unchanged at earlier time points at which histological abnormalities were observed (days 7 and 14). We also failed to observe significant changes in plasma electrolytes prior to day 21 (Supplemental Figure 3), although aldosterone levels were elevated by day 14 (Figure 7B). Because alterations in BUN and creatinine lag behind the onset of renal injury (61), we also assessed 2 early, clinically relevant AKI markers, neutrophil gelatinase-associated lipocalin (NGAL) and kidney injury molecule-1 (KIM-1, also known as HAVCR-1) (62). As anticipated, the messages corresponding to NGAL and KIM-1 (day 14) (Figure 7, C and D) increased prior to the rise in creatinine and BUN (day 21) (Figure 7A) after GRP170 deficiency was established. These data are consistent with severe kidney injury, namely an AKI-like phenomenon, in mice that lack an ER-resident molecular chaperone in the kidney tubule.

### The GRP170-KO mouse exhibits initial signs of kidney failure.

**Figure 8:**
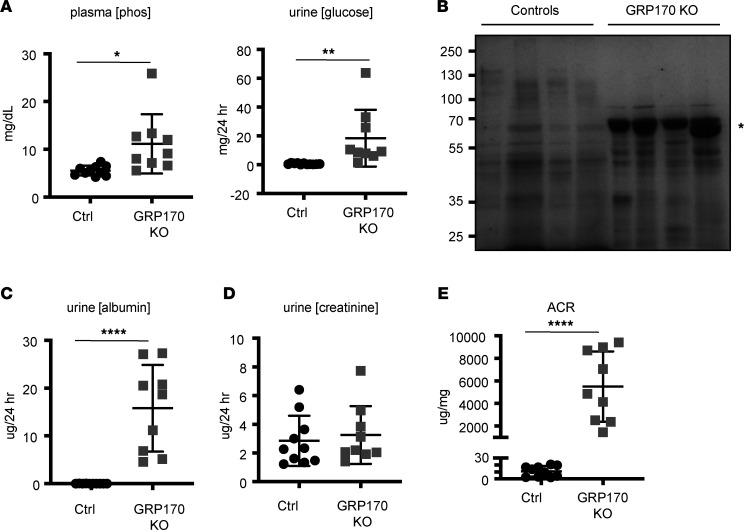
GRP170-KO mice exhibit characteristics of proximal tubule injury. (**A**) Plasma phosphate and urine glucose were measured as described in Methods. (**B** and **C**) Equal volumes of urine from control or GRP170-KO mice (day 21) were subject to SDS-PAGE and stained with Coomassie blue to detect protein as described in Methods. Asterisk denotes molecular weight of albumin. (**D** and **E**) Urine creatinine and the albumin/creatinine ratio (ACR) (all day 21) were determined as described in the Methods. Data are presented as the mean ± SD; *n* = 9–12. *P < 0.05, **P < 0.01, ****P < 0.0001. Statistical significance determined by 2-tailed Student’s *t* test. Data presented are the means ± SD.

Given the rise in plasma AKI markers, evidence of PT dysfunction, and altered histology, we next examined metabolic indicators of renal failure and CKD. In cases of profound renal damage, plasma potassium and phosphorous tend to accumulate due to decreased glomerular filtration rate (GFR) (63). As shown in Figure 2 and Figure 8A, this effect was observed in the GRP170-KO animals at day 21. The GRP170-KO mice also had significantly elevated urine glucose levels (glucosuria), a hallmark of PT injury (Figure 8A). Albuminuria, another marker of proximal tubular injury and common feature of AKI (as well as a risk factor for CKD progression and death), was also evident in the KO mice (64) (Figure 8, B and C), as was an elevated urine albumin/creatinine ratio (ACR) (Figure 8E). In contrast, no difference in 24-hour urine creatinine excretion between the control and GRP170-KO animals was apparent, which is likely a result of the GRP170-deficient mice reaching a new steady state, at which the rates of creatinine production and urinary excretion were equal (Figure 8D).

**Figure 9:**
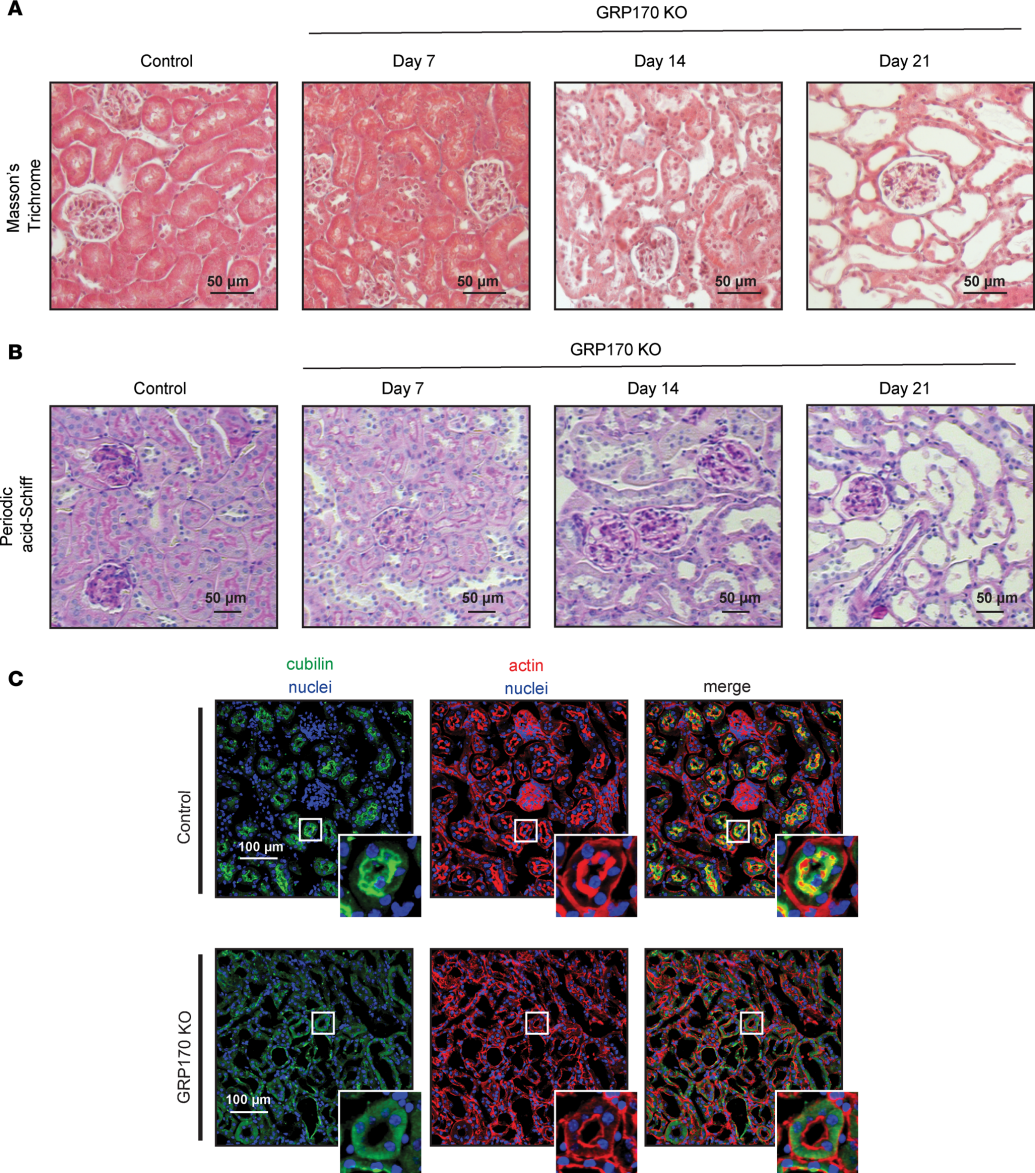
The glomeruli are unaffected by the loss of GRP170 in the nephron. (**A** and **B**) Kidney sections from either control or GRP170-KO mice (day 7, 14, or 21) were subject to processing with either Masson’s trichrome (see boxes on images of full fields Supplemental Figure 2) (**A**) or PAS (**B**) staining. Scale bars: 50 µm. (**C**) Immunofluorescent localization was performed on kidney slices as described in the Supplemental Methods using anti-cubilin antibody or F-actin (phalloidin) as a control. Scale bars: 100 µm.

To distinguish between acute and chronic kidney injury, we next performed Masson’s trichrome and periodic acid–Schiff (PAS) staining, techniques that respectively detect fibrosis and tubular epithelial integrity. Based on prior studies, the duration between initial injury and development of renal fibrosis in mouse models varies, ranging from days (IR injury) to weeks or months, and that in turn depends on underlying conditions (65, 66). In our case (Figure 9A), the GRP170-KO animals exhibited minimal fibrosis, as demonstrated by the absence of any blue signal (Masson’s trichrome), even as late as day 21. PAS staining confirmed the preservation of glomerular architecture, which is typically disrupted in CKD (67) (Figure 9B). These results support our expectation that tubule-specific GRP170 deficiency spares the glomerulus.

Because the endocytic receptor, cubilin (along with megalin) binds to and reabsorbs filtered proteins in the PT, defects in cubilin levels or residence can result in albuminuria (68). Therefore, we assessed cubilin localization in the GRP170-KO mice (Figure 9C). In control animals, cubilin staining lines the apical brush border of PTs. In contrast, in the GRP170-KO mice, cubilin staining was more diffuse and appeared to be more intracellular (see inset panels). We hypothesize that the loss of cubilin at the cell surface explains the proteinuria observed in the GRP170-KO animals and that GRP170 may regulate cubilin biogenesis and trafficking.

### GRP170 deficiency activates the UPR.

Numerous studies have established that a cytoplasmic heat shock response is activated in IR and nephrotoxic injury models (69, 70). For example, a microarray analysis following IR injury in rats identified Hsp70 as the most highly (~43-fold) upregulated gene (71). To assess whether the cellular heat shock response was similarly activated in our model — even though an ER lumenal chaperone is deficient — Hsp70 and Hsp90 expression were assessed by qPCR and Western blot. In contrast to these prior studies, the expression of messages corresponding to these proteins did not increase (Supplemental Figure 4A); in fact, Hsp70 message was diminished at day 21 in kidneys from the KO animal, possibly due to the noted instability of Hsp70 mRNA (72). Similarly, when protein levels were examined, the only effect observed was a minor increase in Hsp90 at day 14 (Supplemental Figure 4B). These data indicate that — despite the devastating cellular injury we observed — the cytoplasmic stress response was not activated.

**Figure 10:**
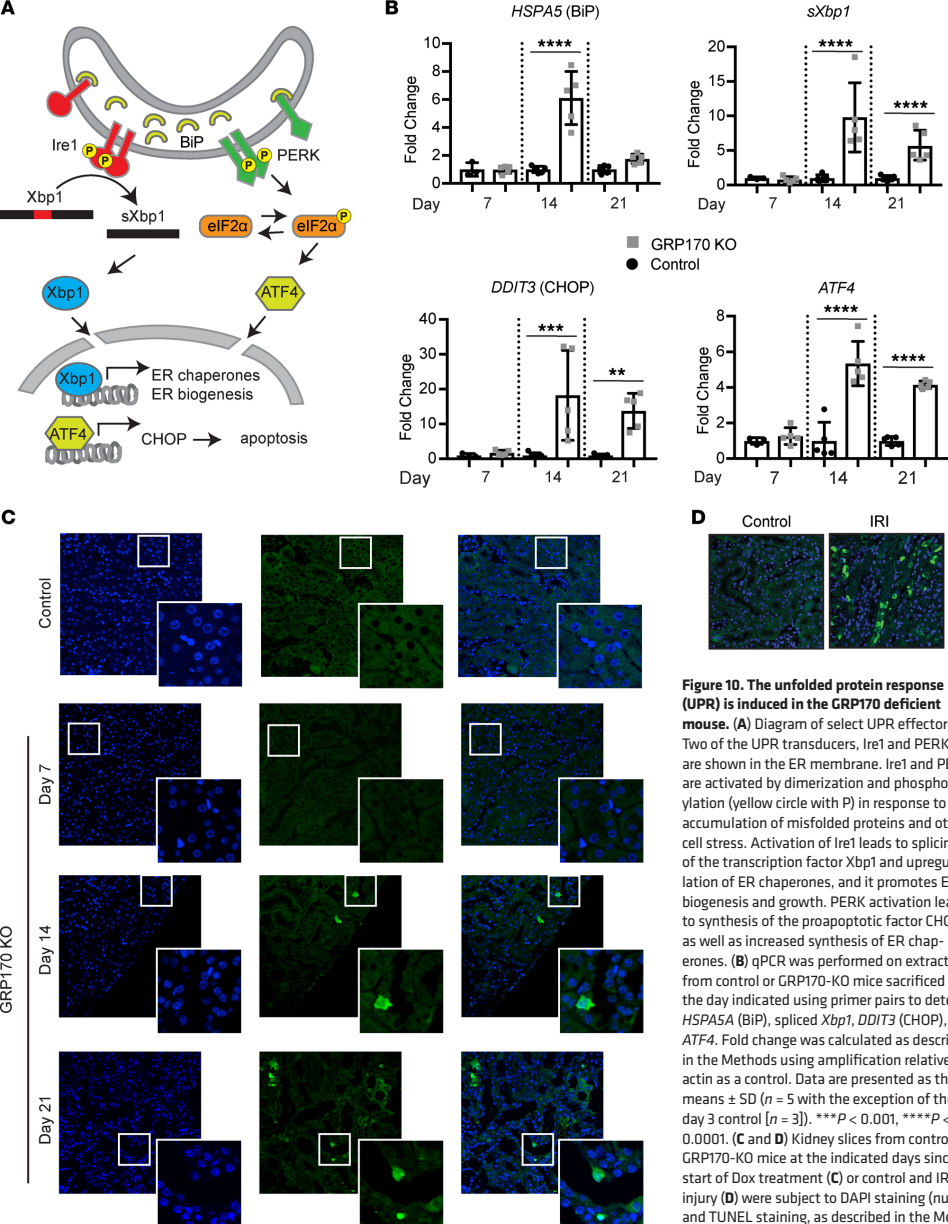
The unfolded protein response (UPR) is induced in the GRP170 deficient mouse. (**A**) Diagram of select UPR effectors. Two of the UPR transducers, Ire1 and PERK, are shown in the ER membrane. Ire1 and PERK are activated by dimerization and phosphorylation (yellow circle with P) in response to accumulation of misfolded proteins and other cell stress. Activation of Ire1 leads to splicing of the transcription factor Xbp1 and upregulation of ER chaperones, and it promotes ER biogenesis and growth. PERK activation leads to synthesis of the proapoptotic factor CHOP, as well as increased synthesis of ER chaperones. (**B**) qPCR was performed on extracts from control or GRP170-KO mice sacrificed at the day indicated using primer pairs to detect *HSPA5A* (BiP), spliced *Xbp1*, *DDIT3* (CHOP), or *ATF4*. Fold change was calculated as described in the Methods using amplification relative to actin as a control. Data are presented as the means ± SD (*n* = 5 with the exception of the day 3 control [*n* = 3]). ***P < 0.001, ****P < 0.0001. (**C** and **D**) Kidney slices from control or GRP170-KO mice at the indicated days since start of Dox treatment (**C**) or control and IR injury (**D**) were subject to DAPI staining (nuclei) and TUNEL staining, as described in the Methods. Representative images are shown. Each unzoomed panel represents an area of 316 µm x 316 µm. Statistical significance determined by 1-way ANOVA followed by Tukey’s multiple-comparison test.

Because GRP170 aids in the folding or degradation of immature proteins in the ER (8, 25, 73, 74) and has been implicated in ER stress–related disorders, ranging from diabetic nephropathy to cardiovascular disease (75), we next hypothesized that the loss of this chaperone activates the UPR. As predicted, messages corresponding to established UPR markers (Figure 10A) were present at day 14 (Figure 10B), coinciding with evidence of renal injury (Figure 7 and Supplemental Figure 2). Specifically, the message corresponding to BiP, the ER lumenal Hsp70 with which GRP170 interacts, was the first reported target of the UPR (76–78). Along with this message, the spliced product of XBP1 and the mRNA corresponding to ATF4, which are sentinels for ER stress via the IRE1 and PERK signaling pathways, respectively (79), were activated (Figure 10B). A downstream effector of ATF4, CHOP (27, 80), was also activated. Similar to BiP, an E3 ligase — Hrd1, which is a UPR target — was upregulated at day 14 (Supplemental Figure 5A). In addition, by using a phospho-specific antibody directed against Ire1 and an antibody directed against PERK, followed by treatment with phosphatase (Supplemental Figure 6, A–C), we also confirmed that these 2 UPR sensors were phosphorylated and therefore activated. Moreover, PERK protein expression was higher in the GRP170-KO animals compared with the control and consistent with UPR activation. Because sustained ER stress can lead to changes in ER morphology and an increase in the ratio of ER sheets to tubules (81), we then examined ER morphology in GRP170-KO kidney tubules through the use of a general ER-specific antibody (anti-KDEL). As shown in Supplemental Figure 7A, the ER in KO animals appears as large bright ovals, as opposed to the dispersed pattern of the ER in the control (Supplemental Figure 7A). These ovals are reminiscent of the localization pattern observed for a specific ER resident protein (i.e., Sec61) in response to ER stress (82). Climp63, which regulates ER architecture and maintains ER sheets, was also elevated at days 14 and 21 (Supplemental Figure 7B) (83, 84), suggesting an expansion of the protein synthesis capacity of the ER. Together, these results strongly suggest that GRP170 depletion induces the UPR.

Consistent with these data, the protein levels of the GRP94 and BiP chaperones, which are UPR targets — as well as phosphorylated eIF2 (a downstream effector of PERK) and the proapoptotic factor, CHOP — were all elevated (Supplemental Figure 5, B–E). Notably, previous studies demonstrated that UPR-triggered kidney injury was muted in mice lacking CHOP (85). Therefore, we next asked whether apoptotic nuclei were present in nephron-resident cells from GRP170-deficient animals. As shown in Figure 10C, kidney slices from GRP170-KO mice at both days 14 day 21 contained apoptotic nuclei (TUNEL^+^), in contrast to controls or slices from day 7 KO mice. However, the levels of TUNEL^+^ cells were lower in our model than in samples from a mouse subject to IR injury (Figure 10D). The more modest presence of apoptosis in our model might, therefore, provide a more reasonable system to examine therapeutics to moderate symptoms associated with AKI. More generally, our findings highlight the value of the GRP170-KO model as a possible means to surgically activate an ER — but not cytosolic — stress response in vivo and link the absence of a specific ER chaperone to compromised water and solute homeostasis, renal damage, and disease.

## Discussion

Molecular chaperones, including GRP170, ensure the proper folding, posttranslational modification, and quality control of nascent membrane and secretory proteins, and their roles in maintaining proteostasis are documented in a number of organ systems, from neurons to cardiac myocytes (86–89). However, the contribution of specific chaperones to kidney physiology is largely unstudied. Therefore, we developed an inducible, nephron-specific murine KO model for GRP170. We show that the loss of this ER chaperone results in a profound phenotype that impairs water and electrolyte homeostasis and leads to hypovolemia, prerenal azotemia, PT dilation, loss of the brush border, UPR activation, and terminal AKI. Thus, our work provides a link between the function of a specific ER chaperone, ER proteostasis, salt and water homeostasis, and kidney injury. The development of this model also provides a facile system in which the effects of renal protectants and newly developed drugs that remedy phenotypes associated with AKI and related diseases can be examined.

We previously demonstrated that GRP170 interacts with and regulates the assembly and degradation of unassembled forms of the ENaC α-subunit in *Xenopus* oocytes and cell culture (8, 25). Consistent with these data, we observed decreased γENaC expression in the CD of GRP170-KO mice, and the phenotype of our GRP170-deficient animals resembles that of mice lacking αENaC, with both models mimicking the effects seen in pseudohypoaldosteronism type 1 (PHA1) (56). In each case, there was dramatic weight loss, hemoconcentration, hyperkalemia, and profound elevation of aldosterone — features consistent with hypovolemia. Mice lacking either GRP170 or αENaC also develop hyponatremia with inappropriate natriuresis and an impaired ability to concentrate urine, both of which are markers of defective water and sodium homeostasis. Furthermore, the animals in the current work and those lacking αENaC develop AKI with elevated plasma creatinine. These data suggest that the loss of ENaC may underly the phenotype of the GRP170-KO animal. However, there are important distinctions between these models. First, relative to αENaC-KO mice, the GRP170-KO phenotype is delayed by ~10 days. This delay may arise from the relative stability of the GRP170 protein versus αENaC (unpublished data) (90–92). Second, the αENaC-KO animal displays decreased potassium excretion and develops metabolic acidosis, whereas the GRP170-KO animal does not. Additionally, while plasma creatinine levels are elevated in both models, the levels in the GRP170-deficient animals are > 10-fold those in control mice, but levels in the αENaC KO model were only ~2-fold higher compared with the control. Consequently, the loss of GRP170 results in more significant renal injury. The severity of the GRP170 phenotype — including albuminuria and glucosuria, which are hallmarks of proximal tubular injury — and the progression of injury also hint at a broader role for this chaperone than previously recognized.

The pattern of injury observed in GRP170-KO mice, summarized above, is compatible with downstream effects of impaired ion channel proteostasis and/or a consequence of ER stress. Indeed, the impact of sustained UPR activation may be independent of, occur in parallel with, or be triggered by hypovolemia-induced (i.e., prerenal) kidney injury attributable to ENaC and/or the dysregulation of other channels. Based on our data, we suggest that GRP170 deficiency results in sustained UPR activation along the length of the nephron. Initially, PT damage is evident, but ultimately widespread kidney injury occurs. This theory is consistent with the subtle histologic changes to the PT well in advance of elevated KIM-1, BUN, and creatinine.

Susceptibility to AKI varies by sex, and in both humans and rodents, males are more susceptible to kidney injury (93–96). Sorting the data by sex confirmed that the male GRP170-KO mice do, in fact, display a more extreme phenotype (Supplemental Figure 8). In addition, because many forms of AKI, including ischemic AKI, cause an inflammatory response (97), the absence of an inflammatory cell infiltrate in specimens from GRP170-depleted animals suggests a possible role for UPR-triggered apoptotic cell death in kidney injury. In line with this view, recent evidence suggests that apoptosis is a prominent feature of AKI and is potentially even more prominent than necrosis in some circumstances (98–100).

The PT — where damage was first seen and became most pronounced — is highly dependent on aerobic metabolism to reabsorb the vast majority of filtered substrates; there is also a lack of antioxidant and antiapoptotic proteins (101–105). The greater susceptibility of the PT to injury is also consistent with enhanced loss of ion channel and solute transporter expression and trafficking observed in this segment compared with the more distal nephron. Consistent with UPR-initiated damage to the PT, constitutive expression of 2 markers measured in this study, ATF4 and CHOP, results in ATP depletion, depressed protein synthesis, oxidative injury, and ultimately apoptosis (80). ATF4 and CHOP are downstream effectors of PERK, an integral membrane UPR transducer (Figure 10A). If cellular stress remains unresolved, PERK triggers CHOP-dependent apoptosis, while the other 2 UPR transducers, IRE1 and ATF6, are suppressed (27, 79, 106). Hence, the same conditions that predispose the PT to prerenal injury may also predispose cells in this segment to UPR-induced renal injury.

In addition to an apparent link between chaperone-regulated proteostasis, the UPR, and renal cell apoptosis observed in this study, chronic proteinuria also induces the UPR. Interestingly, chronic proteinuria, as well as the treatment of immortalized, PT cells with albumin in vitro, upregulates GRP170 expression (107). Proteinuria-induced apoptosis of PT cells also plays a key role in tubulointerstitial injury, eventually leading to CHOP-induced apoptosis (30). Mislocalization of the endoycytic receptors megalin and cubilin in the GRP170-KO mice may also contribute to proteinuria-induced injury and UPR induction. Importantly, kidney biopsies from nephrotic patients showed that, regardless of the origin of kidney injury, the patients had significantly more apoptotic PT cells and elevated expression of GRP170, BiP, and CHOP than healthy controls (30). Although its widespread toxicity must be considered, the administration of HgCl_2_ also triggers AKI in mice via induction of an ER stress response, an effect that is attenuated by the nonspecific stress inhibitor, 4-PBA (30, 108). Similarly, an ER stress inhibitor, tauroursodeoxycholic acid (TUDCA), protects against tubular necrosis in a murine IR model (109). Finally, a number of widely used nephrotoxic pharmaceuticals — including nonsteroidal antiinflammatory drugs, diuretics, and immunosuppressive agents used in kidney transplant — exert their deleterious effects at least in part by direct or indirect induction of ER stress (110). In the future, these and other clinical and preclinical candidates will be examined in our model and either confirm or refute the link between the UPR and kidney injury.

UPR activation in the kidneys of GRP170-deficient mice is consistent with the residence of this ER chaperone and its role in overseeing the biogenesis of proteins that mature in the ER before entering the secretory pathway (8, 25, 74, 111). In contrast, we failed to observe activation of the cytoplasmic stress response. Nevertheless, downstream components of the cytoplasmic stress response also play an important role in maintaining renal cell homeostasis. For example, HSP90 protects tubular cells during the regenerative phase following ischemic and nephrotoxic AKI (112–115). The cytosolic HSP70 chaperone is also induced after IR-related kidney injury (116, 117) and plays a protective role in kidney injury (118, 119) — for example, limiting damage due to oxidative stress (120, 121). Thus, our work and these previously published studies indicate that renal cell homeostasis is sensitive to both ER and cytoplasmic stress.

Although unmitigated UPR induction leads to cell death, organ failure, and disease-like outcomes — as evidenced in our work — it is also important to emphasize that the UPR protects against toxic insults, at least initially. For example, nephron-specific loss of another ER resident — SEC63, which interacts and functions with BiP (122) — causes renal cyst proliferation in a murine autosomal dominant polycystic liver disease (ADPLD) model (123, 124). Moreover, elimination of both SEC63 and components of the protective IRE1 branch of the UPR lead to more significant kidney damage, suggesting that the UPR is protective in the context of SEC63 deficiency (123, 124). Interestingly, deletion of both Ire1 and the GRP170 homolog, Lhs1, in yeast leads to a synthetic lethal phenotype, again consistent with a protective role of the IRE1 branch of the UPR when ER proteostasis is compromised (22). Therefore, it is possible that the UPR is protective in the context of GRP170 KO, at least initially, and that inhibiting the UPR results in a more severe and more rapidly occurring AKI-like phenotype. In either case, our model provides a unique opportunity to investigate how modulation of the ER stress response can be harnessed to explore the mechanisms underlying AKI and, thereby, to identify potential therapeutic targets.

In conclusion, via the generation of a nephron-specific, inducible, molecular chaperone–KO mouse, we provide the first demonstration to our knowledge that ER proteostasis is required for salt and water homeostasis and renal function. Ultimately, the loss of GRP170 in the nephron progresses to terminal AKI, which is preceded by UPR induction. Future studies will determine to what degree mislocalization of ion channels and transporters versus activation of the UPR contribute to the GRP170-KO mouse phenotype. Because elevated levels of UPR targets are observed in kidney injury models and in patients with AKI and CKD (30, 108, 109, 125), we propose that the induction of these genes may serve as an early biomarker for renal disease. Moreover, we propose that our GRP170-deficient mouse will serve as a regulated, inducible model for kidney injury, one that can be used to examine preclinical therapeutics and genetic modifiers of AKI.

## Methods

### Mice.

All experiments were performed using C57BL/6 mice of both sexes that were 14–20 weeks old. Animals were fed standard chow (Prolab Isopro RMH 3000). Animals were housed with a standard 12-hour light/dark cycle.

### Induction of the GRP170-KO, blood chemistry, and metabolic cage measurements.

Cre recombinase expression was induced in GRP170-KO mice and double transgenic littermate (GRP170^fl/fl^/*Pax8* or GRP170^fl/fl^/LC-1) controls by providing drinking water containing 2 g/L Dox (MilliporeSigma) and 5% sucrose (Thermo Fisher Scientific) for 10 days unless otherwise noted (Figure 1C). Sex was noted for each experiment, but owing to the limited number of animals, males and females were combined for statistical analysis (see Supplemental Figure 8 for data separated by sex). Animal weights were initially monitored every 2–3 days until 1 week prior to sacrifice, when daily measurements were taken (21 day mice). Mice were anesthetized with isoflurane, and whole blood was collected by cardiac puncture with a heparinized syringe and analyzed with an iSTAT analyzer (Chem 8 cartridges, Abbot) to determine Na^+^, K^+^, Cl^–^, Ca^+2^, totalCO_2_ (tCO_2_), and BUN. Kidneys, livers, and lungs were harvested and snap frozen in liquid nitrogen or fixed in either 10% formalin for IHC or 4% paraformaldehyde for immunofluorescence. Animals were euthanized by cervical dislocation. Plasma and urine creatinine values were determined using capillary electrophoresis (O’Brien Kidney Core Center at the University of Texas Southwestern Medical Center, Dallas, Texas, USA). The limit of detection for plasma creatine is 0.15 mg/DL and urine is 3.2 mg/DL, based on the level of detection per UT SW Kidney Core that performed the analysis. Therefore, values under the limit of detection are plotted as 0.15 mg/DL and 3.2 mg/DL, respectively, and any subsequent calculations are described as “estimated values.”

Plasma aldosterone levels were assayed using Enzo Life Sciences Aldosterone ELISA kit (ADI-900-173) according to the manufacturer’s protocols. Samples were diluted 20-fold in sample buffer/assay buffer prior to assay. Urine albumin levels were assessed using the ELISA Starter Accessory kit (Bethyl Laboratories, E101), goat anti–mouse albumin antibody (Bethyl Laboratories, E90-134A), goat anti–mouse albumin antibody HRP conjugated (Bethyl Laboratories, E90-134P) and mouse albumin reference serum (ICL Lab, RS-90AL) according to the manufacturers’ outlined protocols. Urine samples were diluted 25- and 2,500-fold, and those from experimental animals were diluted 12,500- and 25,000-fold in water prior to the experiment. The HRP-conjugated secondary antibody (A90-134P) was diluted 120,000-fold. The standard curve was fitted using linear/sigmoidal fit in GraphPad Prism. Reported value for each control animal is the average of 2 measurements of the 25-fold diluted sample; reported value for each experimental animal is the average of measurements of 12,500-fold and 25,000-fold dilutions, each of which is the average of 2 individual measurements at the corresponding concentration.

To determine urine output and water consumption, mice were housed in individual metabolic cages (Tecniplast) and fed a gel-based chow (125 g mouse chow, 250 mL water, and 3 g agar; Prolab Isopro RMH 3000, LabDiets) to reduce contamination of urine samples. Mice were acclimatized to the cages for 1–2 days prior to data collection, and daily weights were taken. Urine volume and volume of water consumed over a 24-hour period was recorded. Urine osmolality was determined using an osmometer (Precision Systems), and urine sodium and potassium concentrations were determined by flame photometry.

### Gene expression studies.

Total RNA was isolated from 25 mg of a whole kidney specimen using manual homogenization with a 25-gauge needle in RLT buffer (RNeasy Mini Kit Qiagen). RNA purity was assessed using a spectrophotometer (Thermo Fisher Scientific, NanoDrop One Microvolume UV-Vis Spectrophotometer) and via agarose gel electrophoresis. For each sample, cDNA was amplified from 1 μg of total RNA per manufacturer’s instructions using QuantaBio qScript Supermix. The quality and quantity of the resulting cDNA was confirmed by spectrophotometry and then diluted to a concentration of 15 ng/μL. qPCR (QuantStudio III) with 80 ng of cDNA per replicate was performed using SYBR green master mix (Applied Biosystems) and the Quantstudio III qPCR system (Thermo Fisher Scientific). After assessing primer efficiency, each primer pair (Supplemental Table 1) was diluted to 25 μM/μL in DNase-free water. Relative amplification was determined from the average of at least 3 technical and 3–5 biological replicates. Gene expression was normalized to β-actin. Fold change in gene expression was calculated using the 2^–ΔΔCt2^ method (126).

### TUNEL assay.

Apoptosis was detected using the ApoTag Fluorescein In Situ Apoptosis Detection Kit S7110 (MilliporeSigma) according to manufacturer’s instructions. Paraffin sections were deparaffinized in xylene and rehydrated with ethanol. Proteinase K (20 μg/mL) was applied to each slide, and samples were incubated for 15 minutes at room temperature. Slides were treated with TdT Enzyme for 1 hour and incubated in the Anti-Digoxin Conjugate antibody solution (MilliporeSigma) for 30 minutes. Slides were counterstained with DAPI (1 μg/mL) and visualized by fluorescence microscopy.

### Histopathology.

For histology, kidneys were fixed overnight in 10% neutral-buffered formalin and then transferred to 70% (v/v) ethanol for storage. Samples were embedded in paraffin, and 10 μm sections were cut and stained with H&E. Images were acquired using an HC JC PL APO 20× (NA 0.80; Leica) objective attached to a Leica DM6000 widefield microscope outfitted with a Jenoptik Progres Gryphax color digital camera running GRYPHAX software. Images were stitched together using the photomerge function of Adobe Photoshop CC 2019, contrast corrected, and layed out in Adobe Illustrator CC 2019. For PAS slides were prepared by the Histology Core at UPMC Children’s Hospital of Pittsburgh in the John G. Rangos Sr. Research Center (Pittsburgh, Pennsylvania, USA). Images were taken on a Leica stereoscope.

### Preparation and analysis of renal proteins.

Mouse kidneys were immediately flash frozen in liquid nitrogen. Each half kidney was homogenized using mortar and pestle in 200 μL cold RIPA buffer (10 mM Tris-Cl [pH 8.0], 140 mM NaCl, 1 mM EDTA, 1% Triton-X 100, 0.1% sodium deoxycholate, 0.1% SDS) with freshly added cOmplete Mini EDTA-free Protease Inhibitor Cocktail (1 tablet/5 mL buffer) (Roche, 4693159001), 1 mM PMSF, 2 μM leupeptin, and 0.7 μM pepstatin A. After the homogenate was centrifuged at 18,000*g* for 10 minutes at 4°C, the supernatant was frozen in liquid nitrogen. Protein concentration was assessed using the BCA colorimetric assay according to the manufacturer’s instructions (Pierce BCA Protein Assay Kit, Thermo Fisher Scientific, 23225). The 5× SDS sample buffer (50% glycerol, 10% SDS, 0.325 M Tris, 0.25 mg/mL bromophenol blue, 5% β-mercaptoethanol [pH 6.8]; MilliporeSigma) was added, and samples were incubated at either 37°C for 30 minutes or 100°C for 5 minutes prior to SDS-PAGE and Western blotting. See Supplemental Table 2 for a complete list of antibodies and conditions used in this study. Secondary antibodies were used at a dilution of 1:10,000 (anti-mouse; Cell Signaling Technologies, 7076S) or anti-rabbit (Cell Signaling Technologies, 7074S). Images were detected on a Bio-Rad Universal Hood II Imager and quantified using ImageJ 1.51h software (NIH).

To detect BiP, 100 μg of samples from control animals and 5–50 μg from the GRP170-KO mice were used. Total protein load was assessed using Revert 700 Total Protein Stain Kits for Western Blot Normalization (LI-COR, 926-11010). Membranes were incubated in Intercept (TBS) Blocking Buffer (LI-COR, 927-60001) and then probed with anti-BiP antibody (127) (see Supplemental Table 2 for antibody information) followed by a 1:10,000 dilution of IRDye 800CW goat anti-rabbit igg secondary antibody (LI-COR, 926-32211). Imaging was performed on a LI-COR Odyssey CLx Imaging System. Western blots were quantified using ImageJ 1.5h software (NIH), and the level of each protein was normalized to either total protein or GAPDH levels, as indicated.

### Statistics.

All measurements included in this study are presented as the mean ± SD. When comparing 2 sets of data, a 2-tailed Student’s *t* test was used to determine significance. When comparing 3 or more data sets, a 1-way ANOVA followed by Tukey’s multiple-comparison test was used to determine significance. *P* < 0.05 was assumed to be significant. For select data sets, both pairwise and multifactorial analyses were performed and are noted.

### Study approval.

All procedures using mice followed *Guide for the Care and Use of Laboratory Animals* (National Academies Press, 2011) and were approved by the Institutional Animal Care and Use Committee of the University of Pittsburgh. The animal studies described here were approved by the IACUC at the University of Pittsburgh, protocol no. 21038908.

## Author contributions

TMB, JLB, TRK, LMH, and ARS devised the project; SG generated the GRP170-KO mouse; ARS provided Pax8/LC1 mice; TMB, AWP, ECR, and ALM designed research studies; TMB, AWP, DNN, ALM, SMM, GA, WGR, DRC, and LJN conducted experiments and/or analyzed data; and TMB, AWP, and JLB wrote the manuscript. All authors contributed to both critical discussions and to editing the final manuscript.

## Supplementary Material

Supplemental data

## Version 1 02/01/2022

In-Press Preview

## Version 2 03/08/2022

Electronic publication
