# Supplementary material for: The molecular chaperone GRP170 protects against ER stress and acute kidney injury in mice
Source: JCI Insight. 2022 Mar 8;7(5):e151869. doi: 10.1172/jci.insight.151869 (PMC8983141; doi:10.1172/jci.insight.151869)
Supplement: Supplemental data [file jciinsight-7-151869-s066.pdf]

Supplemental Methods

Generation and genotyping of a Grp170 conditional knockout mouse

The GRP170 gene contains 26 exons and the start codon is in exon 2. A targeting strategy was designed to insert LoxP in intron 1 and 24 using CRISPR/Cas9 technology (1, 2). Cre-mediated recombination of the two LoxP sites results in the deletion of exon 2 to 23, the transcript resulting from splicing of exon 1 into exon 24 has the potential to encode a short peptide of 54 amino acids, 17 random amino acids (out of frame in WT GRP170) and the last 37amino acids of GRP170.

C57BL/6J (The Jackson Laboratory) zygotes were produced by natural mating of superovulated females. The zygotes were injected with a mixture of Cas9 mRNA (100 ng/µl), two Cas9 single guide RNA: Grp170-i1-1 and Grp170-i24-1 sgRNA (50 ng/µl each) and two oligonucleotides ("Ultramer", Integrated DNA Technologies, Inc): GRP170-i1-1-HDR and GRP170-i24-1-HDR (50 µM, each). The Cas9 mRNA and the Cas9 single guide RNA were produced as previously described (1). The Grp170-i1-1 (GGCTAAGGTTACAAGCCGGG) and Grp170-i24-1 (TAAGAAAGTAAGCAACGAGC) sgRNAs targeted the Cas9 nuclease region to regions in introns 1 and 24, the Cas9 induced two double-strand break that were repaired using the oligonucleotides as templates, which resulted in the seamless insertion of LoxP site along with a SalI restriction sites. Each of the oligonucleotides contained the sequence of the LoxP site and the SalI flanked by sequences homologous to the Grp170 gene.

Sequence of the oligonucleotides (5' – 3'): **GRP170-i1-1-HDR** (intron 1, **Sal1,** LoxP):

CTGAATCCATTTCTGGGAGTGGGATCTTCCACCTTCGTCAGGTGAGGTTTAGGTGA

ACATCCACCC**GTCGAC**ATAACTTCGTATAATGTATGCTATACGAAGTTATGGCTTGT AACCTTAGCCTGCACAACGTCTGCGTAGGCAGCGTGGCCCAGGAGTGGGATGGG CAAGGAG; **GRP170-i24-1-HDR** (intron 24, **Sal1,** LoxP)**:** ATCTAGATGAAGTACCAACTCCAGATTCAAGTGAGAGACCTTCCCCAAATATATAA GAAAGTAAGCAAC**GTCGAC**ATAACTTCGTATAATGTATGCTATACGAAGTTATAGC CGGGTGTGATGGCGCACACCTTTAATCCCAGCACTTGGGAGGCAGAGGCAGGTCT TGTCTACA.

PCR genotyping (see below) identified a founder mouse with correctly inserted LoxP sites in intron 1 and 24, as confirmed by TOPO cloning and sequencing. Co-inheritance of LoxP sites was confirmed by back-crossing the founder to C57/BL6 mice and genotyping the F1 offspring. Next, F1 GRP170-flox heterozygotes were crossed to WT C57/BL6 mice to increase the colony and reduce potential off-target genomic effects resulting from CRISPR/Cas9 editing. The GRP170$^{fl/fl}$ strain will be available at The Jackson Laboratory Repository with the Jax Stock No. 036724 (http://jaxmice.jax.org/query). The resulting confirmed GRP170$^{fl/fl}$ mice were next crossed to the *Pax8*-rtTA/LC-1 mouse (3) to create triple-transgenic GRP170$^{fl/-}$/*Pax8*/LC-1 mice that were then crossed to GRP170$^{fl/fl}$ animals. The triple transgenic mouse was confirmed by PCR genotyping from mice tail samples.

Mouse genotyping was performed as described (4) using the HotSHOT method. In brief, genomic DNA was extracted from a tail snip (<0.2 mm) by incubating the tissue at 95 °C for 45 min in 75 µl reagent 1 (25 mM NaOH, 0.2 mM disodium EDTA, pH 12) followed by the addition of 75 µl reagent 2 (400 mM Tris-HCl). PCR was performed using standard

protocols and following manufactures instructions using Dreamtaq green PCR mastermix (Thermo Fisher Scientific), 2-3 µl of DNA extract per 30 µl PCR reaction, and the appropriate primer pair. Please see Supplemental Table 1 for a complete list of the primers used in this study.

Immunofluorescence and confocal image analysis

For immunofluorescence analysis, kidneys were fixed overnight in 4% paraformaldehyde in PBS at 4°C, and then washed 3 times for 15 min each with cold PBS. Fixation was quenched with 0.2 M ammonium chloride for 1 min followed by three 15 min washes in PBS. Tissue was stored in PBS plus 0.025% (w/v) azide at 4°C. Kidneys were cut in two along their sagittal, median plane and placed cut side down in Tissue-Tek 10mm x 10mm x 5mm cryomolds (Sakura Finetek USA, Torrance, CA) filled with Optimal Cutting Temperature freezing medium (OCT; Miles Inc., Elkhart, IN). The tissue blocks were frozen on dry ice and stored at -80˚ C. Tissue was sectioned at 4 µm using a Leica Microsystems CM1950 cryostat (Buffalo Grove, IL; 5-10 µm sections), and cryosections collected on Superfrost Plus glass slides (ThermoFisher Scientific, Pittsburgh, PA). Unreacted fixative was quenched with Quench Buffer (75 mM NH_4Cl and 20 mM glycine, pH 8.0 dissolved in PBS, containing 0.1% v/v Triton X-100) for 10 min at RT (room temperature). The tissue was rinsed three times with PBS, and then incubated in Block Solution (PBS containing 0.6% v/v fish skin gelatin, 0.05% w/v saponin) supplemented with 5% v/v donkey serum for 60 min at RT. Primary antibodies, diluted in Block Solution, were incubated for 2 hr at RT or overnight at 4 °C in a humid chamber. The slides were washed 3-times quickly and 3 times for 5 min with Block

Solution, and then incubated with minimal cross reactivity, fluorophore-labeled secondary antibodies, diluted in Block Solution, for 1 hr at room temperature. Nuclei were counterstained with TO-PRO-3 (1:1000; ThermoFisher Scientific) and overall tissue architecture visualized using tetramethylrhodamine isothiocyanate (TRITC)–labeled phalloidin (ThermoFisher Scientific; 1:200). The labeled tissues were then rinsed 3 times quickly and 3 times for 5 min with Block Solution, rinsed with PBS, and postfixed in 4% paraformaldehyde (dissolved in 100 mM phosphate buffer, pH 7.4) for 5 min at RT. The slides were rinsed with PBS, a drop of SlowFade Diamond Antifade (Molecular Probes - ThermoFisher) was placed on the tissue, and tissue mounted under borosilicate coverslips (#1.5, 0.17 mm thickness, 24 x 50 mm; ThermoFisher). The edges of the coverslip were sealed with clear nail polish, and the slides stored at -20 °C until image acquisition was performed.

Images were captured using a Leica HCX PL APO CS 40X, 1.25 NA oil objective and the appropriate laser lines of a Leica TCS SP5 CW-STED confocal microscope (in normal confocal mode). The HyD detectors were optimized using the Q-LUT option, and 8-bit images collected using 8 line averages combined with 4 frame averages. Cross-talk between channels was prevented by use of spectral detectors coupled with sequential scanning. Image stacks (1024 x 1024, 8-bit, 3-6 images) were collected using a Z-step of 0.29 µm. Image were imported into Volocity 4-D software (Perkin Elmers; Waltham, MA), and following image reconstruction, exported as TIFF files. The contrast was corrected in Photoshop CC2019 (Adobe; San Jose, CA), and composite

images prepared in Adobe Illustrator CC2019. Representative images are shown in each figure.

See Table S2 for primary antibodies and conditions used for immunofluorescence experiments. Minimal cross-reacting goat or donkey secondary antibodies, conjugated to Alexa488 or CY3, were purchased from Jackson ImmunoResearch Laboratories Inc. (Westgrove, PA).

1. Pelletier S, Gingras S, and Green DR. Mouse genome engineering via CRISPR-Cas9 for study of immune function. *Immunity.* 2015;42(1):18-27.
2. Yang H, Wang H, Shivalila CS, Cheng AW, Shi L, and Jaenisch R. One-step generation of mice carrying reporter and conditional alleles by CRISPR/Cas-mediated genome engineering. *Cell.* 2013;154(6):1370-9.
3. Traykova-Brauch M, Schonig K, Greiner O, Miloud T, Jauch A, Bode M, et al. An efficient and versatile system for acute and chronic modulation of renal tubular function in transgenic mice. *Nat Med.* 2008;14(9):979-84.
4. Truett GE, Heeger P, Mynatt RL, Truett AA, Walker JA, and Warman ML. Preparation of PCR-quality mouse genomic DNA with hot sodium hydroxide and tris (HotSHOT). *Biotechniques.* 2000;29(1):52, 4.

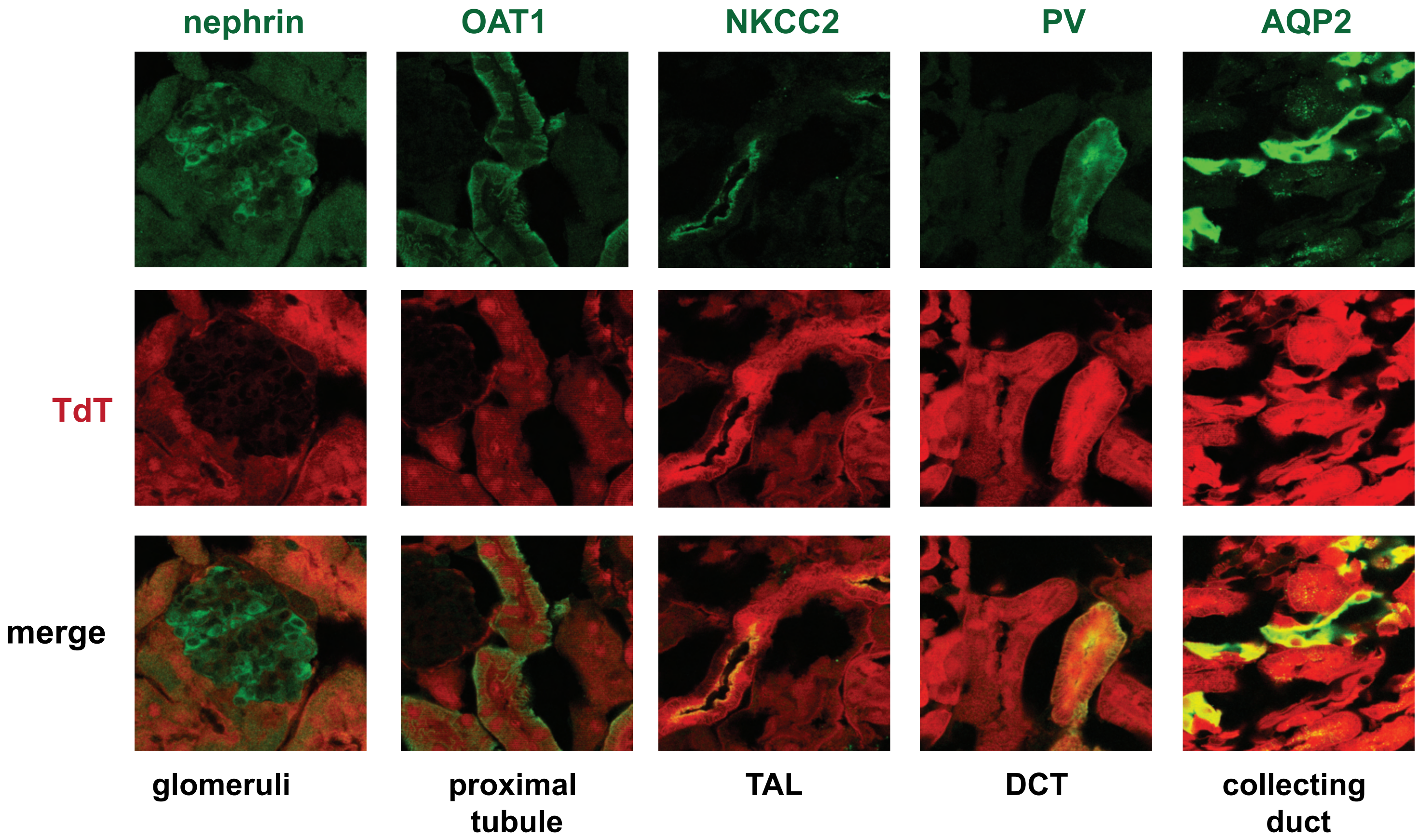

Figure S1. Cre recombinase is expressed in all kidney tubule segments. The Pax8/LC1 mice were crossed to a reporter mouse strain, Rosa26/CAGTdTomato, to assess Cre-recombinase expression. Immunofluorescent localization was performed at day 21 as described in Methods using the following antibodies to detect markers of various kidney segments: glomerulus (anti-nephrin), proximal tubule (anti-OAT1), thick ascending limb of loop of Henle (TAL) (anti-NKCC2), distal convoluted tubuel (DCT) (anti-parvalbumin) and the collecting duct (anti-AQP2).

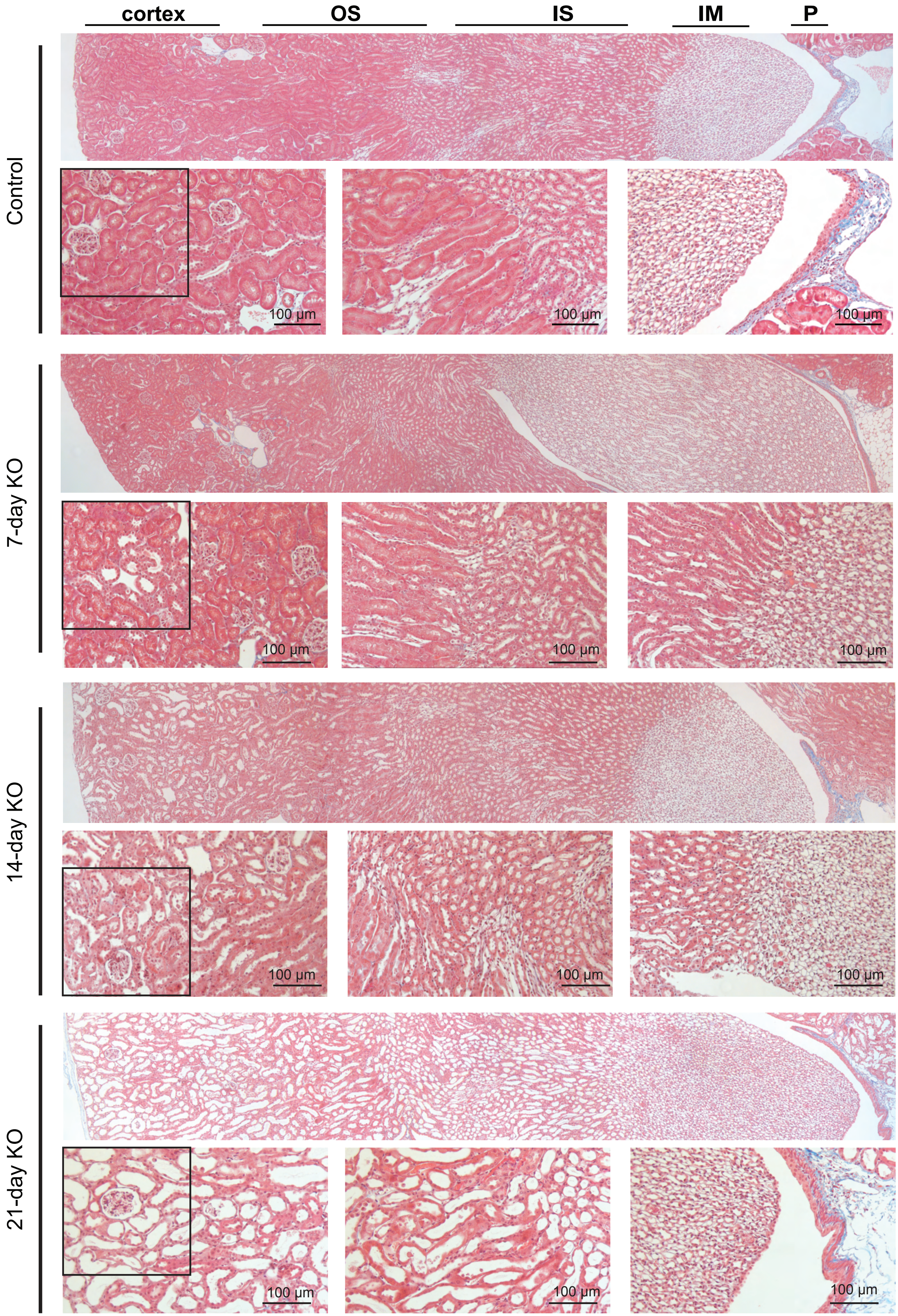

Figure S2. Loss of GRP170 results in progressive kidney injury. Masson's Trichrome staining of fixed kidney slices from either control or GRP170 KO animals harvested at day 7, 14 or 21 from the start of doxycycline administration. Control animals are "day 21." Boxes represent data used for Figure 9A.

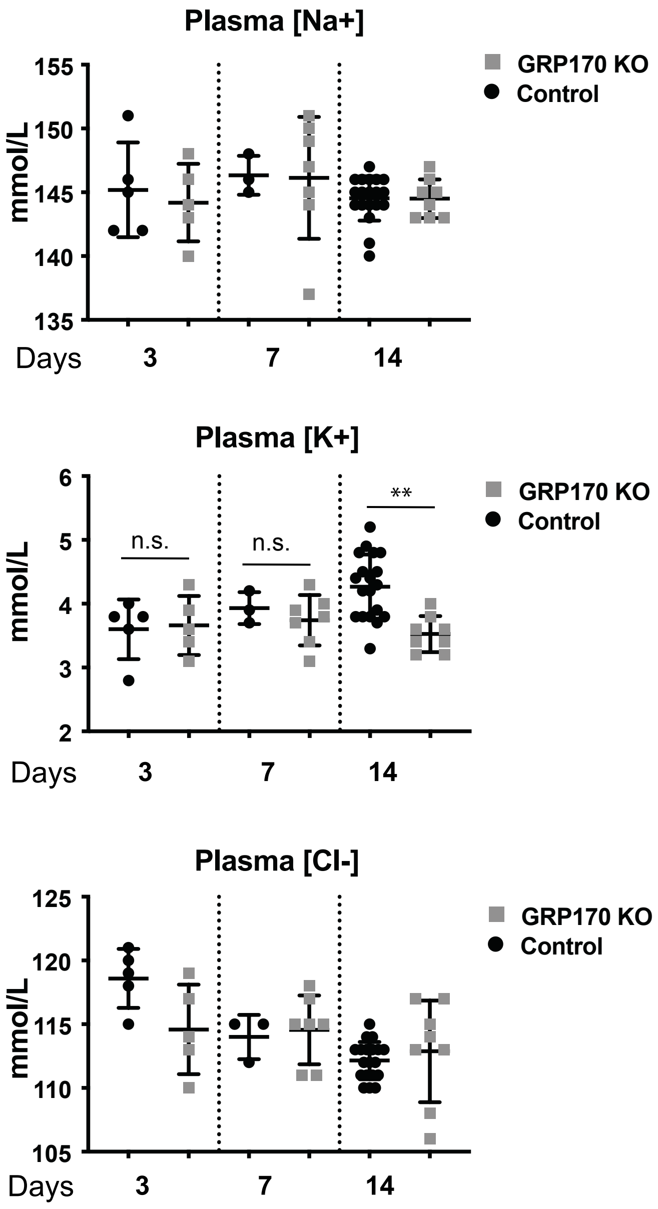

Figure S3. Plasma electrolytes. Plasma electrolytes, sodium, potassium and chloride were measured on day 3, 7 or 14 following dox administration as described in the Methods from control and GRP170 KO mice. Data represent the means +/-SD; n=5-19 with exception of day 7 control (n=3); **p<0.01.

A

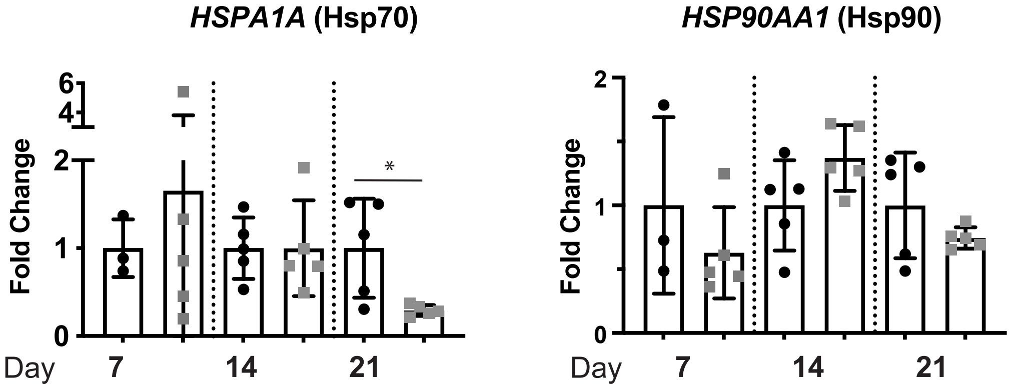

B

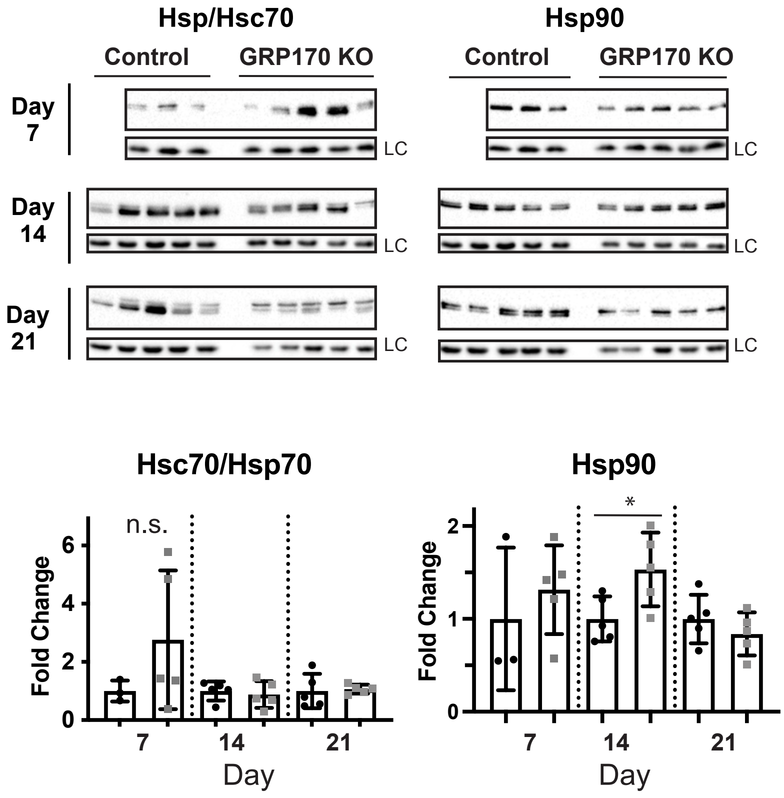

Figure S4. Loss of GRP170 does not result in a heat shock response. (A) Quantitative PCR was performed on extracts from control or GRP170 KO mice sacrificed at day 7, 14 or 21 using primer pairs to detect HSPA1A (Hsp70) or HSP90AA1 (Hsp90). Fold change was calculated as described in Methods using amplification relative to actin as a control. (B) Whole kidney lysates were prepared and subject to SDS-PAGE and western blotting as described in Methods. Western blots were probed with anti-Hsp70 or anti-Hsp90 antibody as well as GAPDH as a loading control (LC). Data are presented as the means +/-SD (n=5 with the exception of the day 3 control n=3); *$p<0.05$.

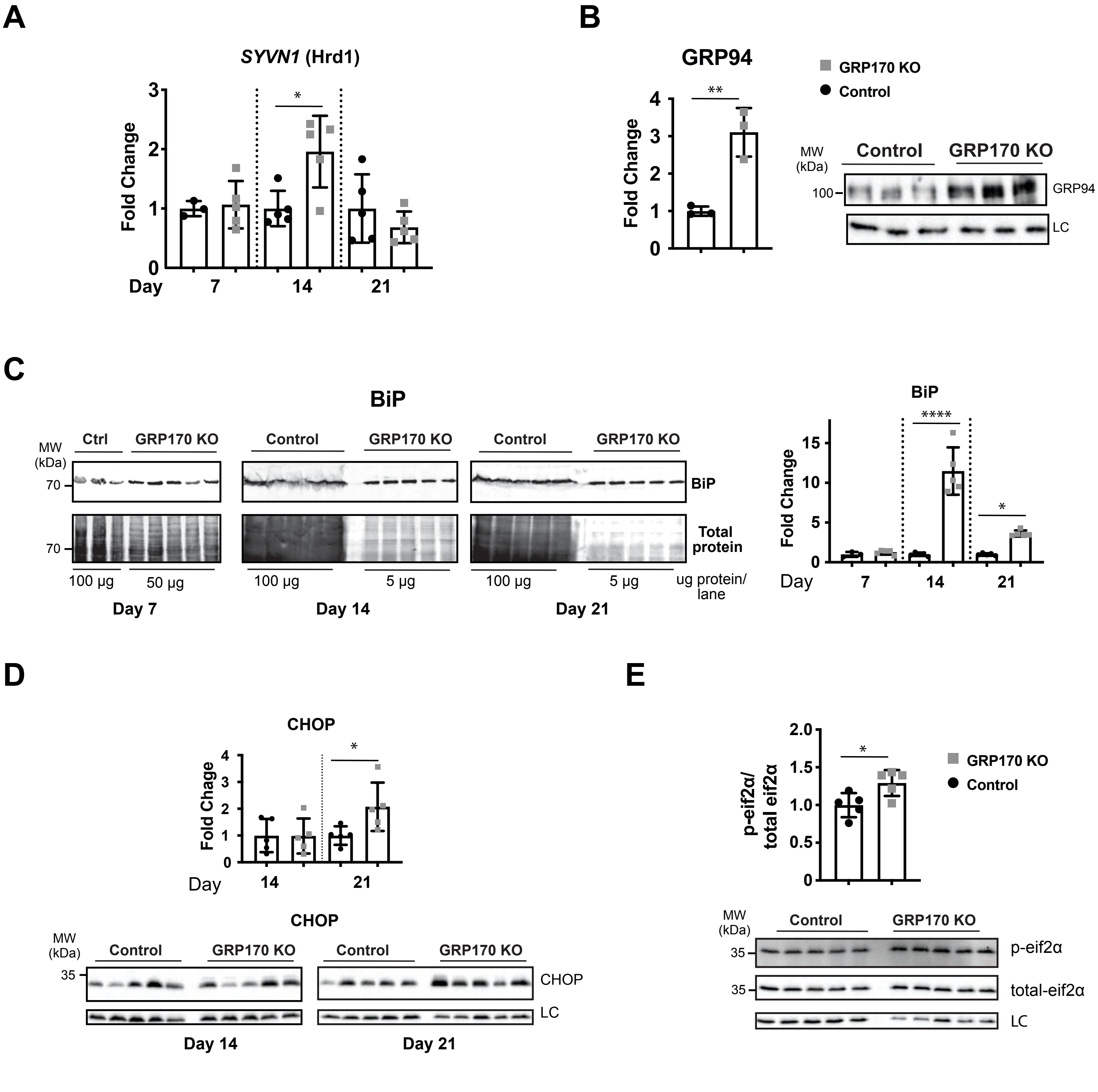

Figure S5. Loss of GRP170 results in induction of the unfolded protein response (UPR). (A) Quantitative PCR was performed on kidney lysates as described in Methods to detect *SYNV1* (Hrd1) and *SEL1L.* Data are corrected to actin mRNA and represent the means +/-SD; n=3-5. Whole kidney lysates were prepared and subject to SDS-PAGE and western blotting as described in Methods. Western blots were probed with (B) anti-GRP94 as well as anti-GAPDH as a loading control (LC) (C) anti-BiP antibody as well as a total protein stain as a loading control, (D) anti-CHOP as well as anti-GAPDH as a loading control (LC), and (E) anti-phosphorylated eIF2α (p-eif2α), anti-eIF2α (eif2α), and anti-GAPDH as a loading control (LC). Data are presented as the means +/-SD (n=5 with the exception of the day 3 control n=3); *p<0.05, **p<0.01, ***p<0.001.

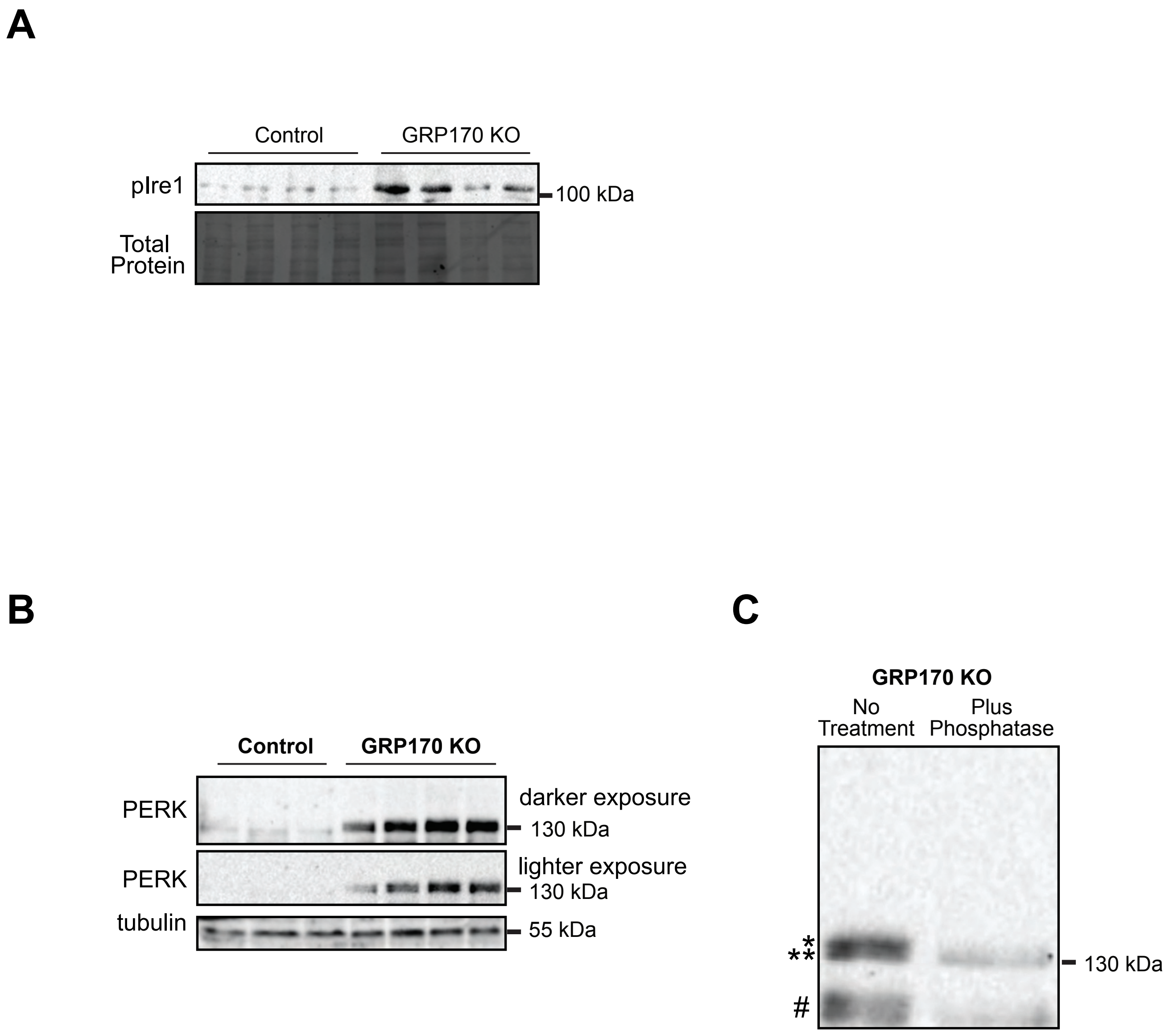

Figure S6. The UPR sensors PERK and Ire1 are activated in the GRP170 KO. (A) Whole kidney lysates from control or GRP170 KO mice (day 21) were prepared and subject to SDS-PAGE and western blotting as described in Methods. Western blots were probed with (A) anti-pIre1 to detected phosporylated Ire1 or (B) anti-PERK as well as anti-tubulin or stained with Revert 700 (LI-COR) total protein stain as a loading control. (C) Kidney lysates were treated with lamda protein phosphatase (New England Bio Labs) according to manufacturer's instructions and then subject to western blotting. Phosphorylated PERK (*) and dephosphorylated PERK (**) were detected as well as degradation products (#) that also demonstrated a shift in molecular weight upon phosphatase treatment. Representative blots are shown for (A) and (B), and each lane respresents an individual mouse.

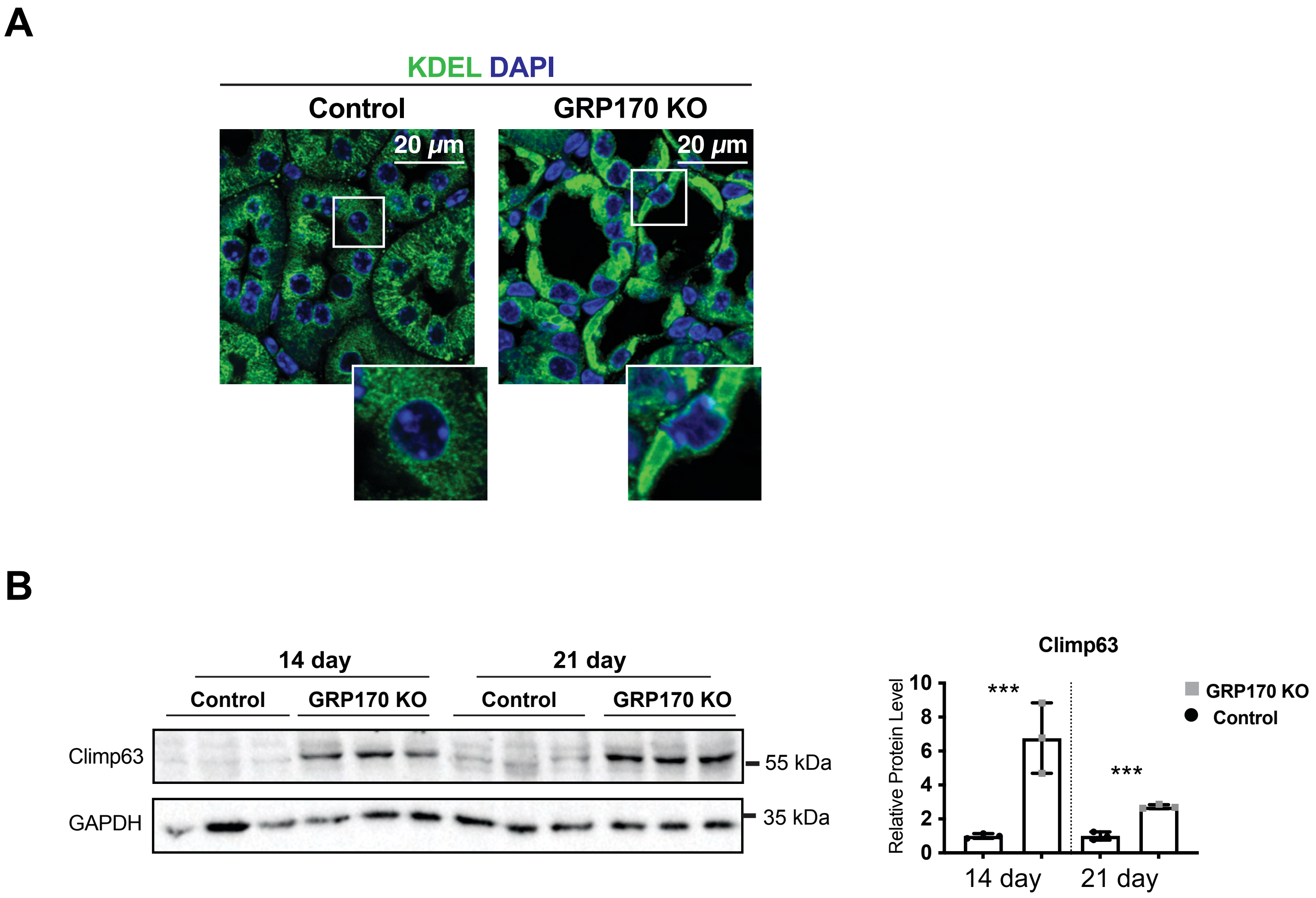

Figure S7. Loss of GRP170 results in an altered ER morphology. (A) Immunofluorescent localization was performed at day 21 as described in Methods using an anti-KDEL antibody to visualize the endoplasmic reticulum (B) Whole kidney lysates were prepared and subject to SDS-PAGE and western blotting as described in Methods. Western blots were probed with anti-Climp63 and GAPDH as a loading control. Data are normalized to control animals and presented as the means +/-SD (n=6); ***p<0.001.

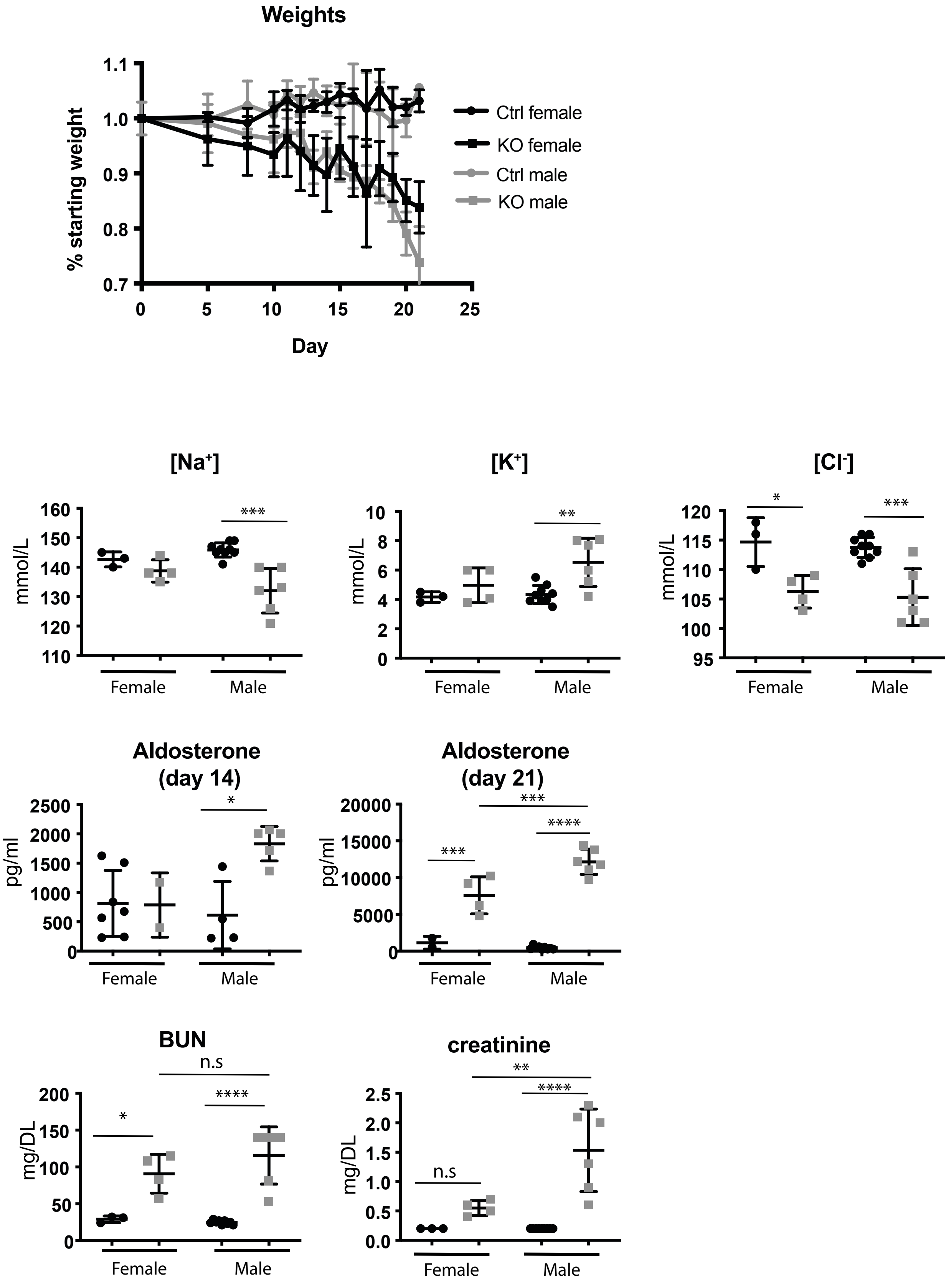

Figure S8. Comparison by sex of GRP170 KO phenotype. Data presented and described in Figures 2 and 7 is separated and plotted based on the sex of control and GRP170 KO mice. Data represent the means +/-SD; n=2-9; *p<0.5, **p<0.01, ***p<0.001, ****p<0.0001.

		Sequence (5'-3')	
	Gene	**Forward**	**Reverse**
qPCR Primer Sequences	*Hsp70*	5'-GCTGAGAGTCGTTGAAGTAGGC-3'	5'-TGGTGCAGTCCGACATGAAG-3'
	Hsp90	5'-GACGCTCTGGATAAAATCCGTT-3'	5'-TGGGAATGAGATTGATGTGCAG-3'
	Kim1	5'-AGCTCAGGGTCTCCTTCACA-3'	5'-ACCACCCCCTTTACTTCCAC-3'
	Ngal	5'-CCATCTATGAGCTACAAGAGAACAAT-3'	5'-TCTGATCCAGTAGCGACAGC-3'
	βENaC	5'-CACCACCTTAGCTGCCATCA-3'	5'-CCCCTCACAGATGATGCGTT-3'
	18s	5'-GTAACCCGTTGAACCCCATT-3'	5'-CCATCCAATCGGTAGTAGCG-3'
	αENaC	5'-TGGATGCCGTGAGAGAATCG-3'	5'-ATGGGGTGGTGGAACTGAGA-3'
	γENaC	5'-GCCGTGACCCTTCAGTTCAG-3'	5'-CTTAATGGTCGGTGCCTGGG-3'
	Aqp1	5'-CCGAGACTTAGGTGGCTCAG-3'	5'-TGATACCGCCAGTGTAG-3'
	Aqp2	5'-CTGTGGAGCTCTTCCTGACC-3'	5'-GGCTACCCAGGTTGTCACTG-3'
	Bip	5'-GGATCATCAATGAGCCTACAGC-3'	5'-ACCCAGGTCAAACACAAGGAT-3'
	Chop	5'-CCTAGCTTGGCTGACAGAGG-3'	5'-CTGCTCCTTCTCCTTCATGC-3'
	Grp170	5'-CCTGAGGATCTTCGGGTATTTG-3'	5'-CTTTGGACTCATAGTCGGGATATT-3'
	Ncc	5'-GTGCCGGCCTACGAACAC-3'	5'-GGTGGCTACCTTCCTGCTTGAG-3'
	Nhe3	5'-CGCACAGAAGCGGAGGAAT-3'	5'-CAAATCTTTCTCCTTGATGGTGTAG-3'
	Nkcc2	5'-GTGGTTATTTGATGATGGAGGGTT-3'	5'-TGCGGTTGATCTTTCCTCCA-3'
	Romk	5'-CGGGAT CCGGCACTGACAGAAAGGATGA-3'	5'-GGAATTCCAGGTCAAGTACAGTTGTCC-3'
	Actinβ	5'-GGCTGTATTCCCCTCCATCG-3'	5'-CCAGTTGGTAACAATGCCATGT-3'
	Xbp1s	5'-GGTCTGCTGAGTCCGCAGCAGG-3'	5'-GAAAGGGAGGCTGGTAAGGAAC-3'
	Atf4	5'-GCAAGGAGGATGCCTTTTC-3'	5'-GTTTCCAGGTCATCCATTCG-3'
	Cre recombinase	5'-CGGTCTGGCAGTAAAAACTAT-3'	5'-CAGGGTGTTATAAGCAATCCC-3'
	Hrd1	5’-AGCTACTTCAGTGAACCCC-3’	5’-TCTACAATGCCCACTGAC-3’
Genotyping Primers	*GRP170_I1*	5'-GAGGATGGAGCAGCCGTC-3'	5'-GACCCTCGAAATCGGCTCAA-3'
	GRP170_I24	5’- CGGTGAATGTGGTGGCCTTT-3’	5’- CCCCGAGACAGGGTTTCTCT-3’
	Pax8	5’-CCATGTCTAGACTGGACAAGA-3’	5’-CAGAAAGTCTTGCCATGACT-3’
	LC-1	5’-TTTCCCGCAGAACCTGAAGATG-3’	5’-TCACCGGCATCAACGTTTTCTT-3’

Table S2

Target	Source	Host	Species reactivity (according to manufacturer)	Application (Dilution)
AQP2 (aquaporin 2)	Alomone AQP-002	rabbit	human, mouse, rat	IF (1:500)
AQP2 (aquaporin 2)	Santa Cruz sc-9882 discontinued	goat	mouse, rat, human	IF (1:1000)
Cubilin (CUBN)	Biorbyt orb4997	rabbit	human, mouse, pig, rat	IF (1:200)
γENaC	StressMarq SPC-405	rabbit	Xenopus, hamster, human, mouse, rat	IF (1:200)
Lotus Tetragonolobus lectin-FITC	Vector Laboratories FL-1321-2	NA	NA	IF (1:500)
NCC (SLC12A3)	StressMarq SPC-402	rabbit	human, mouse, rat, dog	IF (1:200)
NKCC2 (SLC12A1)	StressMarq SPC-401	rabbit	human, mouse, rat	IF (1:200)
NHE3	StressMarq SPC-400D	rabbit	mouse, rat	IF (1:100)
OAT1	Alpha Diagnostics International OAT11-A	rabbit	human, mouse, rat	IF (1:200)
Uromodulin (UMOD)	Thermo Fisher Scientific MA5-24374	rat	mouse	IF (1:200)
BiP	Hendershot et al., 1995	rabbit	N/A	IB (1:1000)
CHOP (LC3F7)	Cell Signaling #2895S	mouse	human, mouse, rat	IB (1:1000)
Hsp70	Cell Signaling #4872S	rabbit	human, mouse, rat, monkey, bovine	IB (1:1000)
GRP170	Behnke et al., 2014	rabbit	N/A	IB (1:1000)
GAPDH (HRP)	Proteintech #HRP-60004	mouse	human, mouse, rat, zebrafish, plant	IB (1:10,000)
Cre Recombinase	Covance #PRB-106C	rabbit		IB (1:1000)
HSP90α	Enzo Life Sciences #ADI-SPS-771-F	rabbit	human, mouse, rat beluga, bovine, dog, fish, guinea pig, hamster, monkey, porcine, rabbit, sheep, Xenopus	IB (1:1000)
eIF2alpha (D7D3) XP(R)	Cell Signaling #5324S	rabbit	human, mouse, rat, monkey	IB (1:1000)
KDEL	Invitrogen #PA1-013	rabbit	hamster, human, mouse, rat	IF (1:500)

Climp63 (CKAP4)	Bethyl Laboratories A302-257A	rabbit	human, mouse	IB (1:1000)
GRP94	Enzo, ADI-SPA-850; 9G10	mouse	human, mouse, rat<br>bovine, chicken, dog, guinea pig, hamster, monkey, porcine, rabbit, sheep, Xenopus	IB (1:1000)
parvalbumin	SWant GP72	Guinea Pig		IF
PERK	Cell Signaling 3192	rabbit	human, mouse, rat, monkey	IB (1:1000)
Phospho-Ire1	AffiniTech AF7510	rabbit	human, mouse, rat	IB (1:1000)
Alpha Tubulin (HRP)	Proteintech 66031	mouse	human, mouse, rat, canine	IB (1:8000)
